# Encapsulation and Biological Activity of Hesperetin Derivatives with HP-β-CD

**DOI:** 10.3390/molecules28196893

**Published:** 2023-09-30

**Authors:** Anna Sykuła, Agnieszka Bodzioch, Adriana Nowak, Waldemar Maniukiewicz, Sylwia Ścieszka, Lidia Piekarska-Radzik, Elżbieta Klewicka, Damian Batory, Elżbieta Łodyga-Chruścińska

**Affiliations:** 1 Faculty of Biotechnology and Food Sciences, Institute of Natural Products and Cosmetics, Lodz University of Technology, Stefanowskiego 2/22, 90-537 Lodz, Poland; anna.sykula@p.lodz.pl; 2 Centre of Molecular and Macromolecular Studies Polish Academy of Sciences, Sienkiewicza 112, 90-363 Lodz, Poland; agnieszka.bodzioch@cbmm.lodz.pl; 3 Department of Environmental Biotechnology, Faculty of Biotechnology and Food Sciences, Lodz University of Technology, Wólczańska 171/173, 90-530 Lodz, Poland; adriana.nowak@p.lodz.pl; 4 Faculty of Chemistry, Institute of General and Ecological Chemistry, Lodz University of Technology, Żeromskiego 116, 90-924 Lodz, Poland; waldemar.maniukiewicz@p.lodz.pl; 5 Institute of Fermentation Technology and Microbiology, Faculty of Biotechnology and Food Sciences, Lodz University of Technology, 90-530 Lodz, Poland; sylwia.scieszka@p.lodz.pl (S.Ś.); lidia.piekarska-radzik@p.lodz.pl (L.P.-R.); elzbieta.klewicka@p.lodz.pl (E.K.); 6 Department of Vehicles and Fundamentals of Machine Design, Lodz University of Technology, 90-924 Lodz, Poland; damian.batory@p.lodz.pl

**Keywords:** hesperetin, flavanone, cyclodextrins, bioavailability, encapsulation

## Abstract

The encapsulation of insoluble compounds can help improve their solubility and activity. The effects of cyclodextrin encapsulation on hesperetin’s derivatives (HHSB, HIN, and HTSC) and the physicochemical properties of the formed complexes were determined using various analytical techniques. The antioxidant (DPPH^•^, ABTS^•+^ scavenging, and Fe^2+^-chelating ability), cytotoxic, and antibacterial activities were also investigated. The inclusion systems were prepared using mechanical and co-evaporation methods using a molar ratio compound: HP-β-CD = 1:1. The identification of solid systems confirmed the formation of two inclusion complexes at hesperetin (CV) and HHSB (mech). The identification of systems of hesperetin and its derivatives with HP-β-CD in solutions at pHs 3.6, 6.5, and 8.5 and at various temperatures (25, 37 and 60 °C) confirmed the effect of cyclodextrin on their solubility. In the DPPH^•^ and ABTS^•+^ assay, pure compounds were characterized by higher antioxidant activity than the complexes. In the FRAP study, all hesperetin and HHSB complexes and HTSC-HP-β-CD (mech) were characterized by higher values of antioxidant activity than pure compounds. The results obtained from cytotoxic activity tests show that for most of the systems tested, cytotoxicity increased with the concentration of the chemical, with the exception of HP-β-CD. All systems inhibited *Escherichia coli* and *Staphylococcus aureus*.

## 1. Introduction

Hesperetin (H) (3′,5,7-Trihydroxy-4′-methoxyflavanone), in the form of aglycone, belongs to the flavanone class of flavonoids. Hesperetin, in the form of its glycoside (hesperidin), is the predominant flavonoid in citrus (family Rutaceae) fruits, such as lemons, limes, mandarins, oranges, and grapefruits. It has been proven that hesperedin is absorbed in the form of hesperetin during digestion in humans and animals.

It has been proven that hesperetin demonstrated antioxidant activity. The compound shows an increased effect on radical scavenging activity in antioxidant assays than its derivatives: hesperidin and hesperidin glucoside (the latter relative to each other show similar antioxidant activity) [1]. An aglycone holds more potent in vitro XO inhibitory activity than the glycosylate derivatives (hesperidin or G-hesperidin). In addition, due to the higher bioavailability of hesperitin, increased SOD activity was observed in the liver of animals [2]. The flavanone also acts in terms of scavenging elevated ROS and enhancing endogenous antioxidant defense mechanisms, mainly by regulating the expression of the transcription factor nuclear factor-2 erythroid-2 (Nrf2) and its target heme oxygenase-1 (HO-1) [3]. Moreover, the supplementation with hesperetin (40 mg/kg, i.p.) to STZ-induced experimental rats for 45 days proved that flavanone can alleviate hyperglycemia and dyslipidemia through enhancing antioxidant competence in STZ-induced experimental rats [4].

Hesperetin is also characterized by cytotoxic activity. For instance, hesperetin remarkably suppressed the cytotoxic efficacy of taxanes in prostate cancer cells. The parallel exposure of cells to hesperetin and taxanes caused a 9.8- and 13.1-fold decrease in the cytotoxicity of docetaxel and cabazitaxel, respectively [5]. Hesperetin can be also used as a potential agent to promote tumor immune response and antigen immunogenicity through increased cytotoxic T lymphocyte (CTL) response and inactivation of B16F10 cells (cells using in skin cancer research) [6]. Additionally, hesperetin decreases the main virulence factors, such as cytotoxin-associated antigen A (CagA) and vacuolytic cytotoxin A (VacA), and diminishes the translocation of CagA and VacA proteins into gastric adenocarcinoma cells (AGS) [1].

Hesperetin has been reported to possess antibacterial effects. For instance, it has an effect against the important human pathogen *Helicobacter pylori*, inhibiting the growth of both reference strains and clinical isolates of the bacterium [7]. Flavanone also demonstrates antimicrobial activity against methicillin-sensitive and -resistant *Staphylococcus aureus* isolates. The minimum inhibitory concentrations for hesperetin were obtained at 250 and 500 g/mL, respectively. The inhibitory effect was reversible using -lactam antibiotics such as methicillin, penicillin, and oxacillin [8]. The study on antibacterial activity proved that hesperetin performed significantly better in terms of inhibiting the bacterial growth, demonstrating MIC values of 125 μg/mL against *Staphylococcus aureus*, 250 μg/mL against *Bacillus cereus*, and 500 μg/mL against *Escherichia coli* and *Pseudomonas aeruginosa* than hesperidin [1].

Schiff bases are of great interest due to the presence of an active linkage (-CO-NHN=C-), which affects their biological activity (anticancer, antifungal, antimicrobial, and many others). Due to their high iron-chelating activity both in vitro and in vivo, some hydrazones have significant effects on cellular iron levels. Therefore, they are used in the treatment of diseases associated with iron overload [9,10,11]. Schiff base can be formed from isoniazid (IN), benzhydrazide (HSB), and thiosemicarbazide (TSC) [12,13,14,15,16,17].

**Figure 1:**
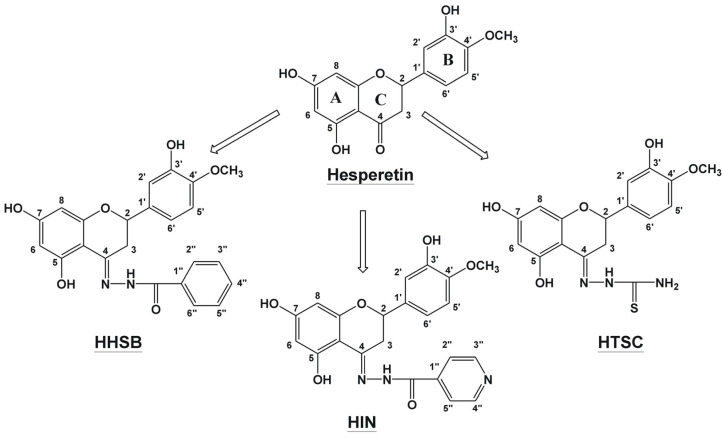
The studied compounds: hesperetin and Schiff bases (HHSB, HIN and HTSC). The **A**, **B** and **C** labels in the hesperetin structure correspond to two aromatic rings (**A** and **B**) connected by a three-carbon α,β-unsaturated carbonyl system (**C**) [18].

Schiff bases are also created on the basis of the hesperetin molecule. Three of them (HHSB, HIN, and HTSC) (Figure 1) were described in our earlier publications [14,18,19]. The derivatives are well soluble in DMSO and DMF, while their solubility in water is unsatisfactory. Therefore, complexing insoluble compounds with cyclodextrins makes it possible, without changing their original and basic structures, to increase their solubility in water, resist factors such as oxygen or light, or dispose of their odor, increasing their bioavailability [20,21,22,23].

Cyclodextrins (CDs) are cyclic oligosaccharides of natural origin; obtained from starch by a simple enzymatic process [24]. They are now produced in large quantities (many thousands of tons per year) using environmentally friendly technologies [25]. Their chemical properties can be easily and significantly modified [26]. Cyclodextrins can be consumed by humans as additives in food, drugs, and cosmetics. Their extremely low toxicity is a result of their metabolism of glucose in vivo. Moreover, they do not accumulate in the body and do not cause local irritation [27]. The use of cyclodextrins occurs mostly as additives in pharmaceutical preparations. It has been found that small amounts of 2-hydroxypropyl-β-cyclodextrin (HP-β-CD) induce a sustaining deployment (parachute effect) on indomethacin [28], and the solubility relationship of the cimetidine/cyclodextrin system relative to its crystal structure [29]. Inclusion complex systems in drugs are the subject of much research work due to the improvement of the physicochemical properties (solubility, dissolution rate, and stability) of pharmaceutical preparations [23]. In an inclusion complex, guest molecules can be placed in the cavities of cyclodextrins (host), and in the intermolecular spaces formed by the hosts.

**Figure 2:**
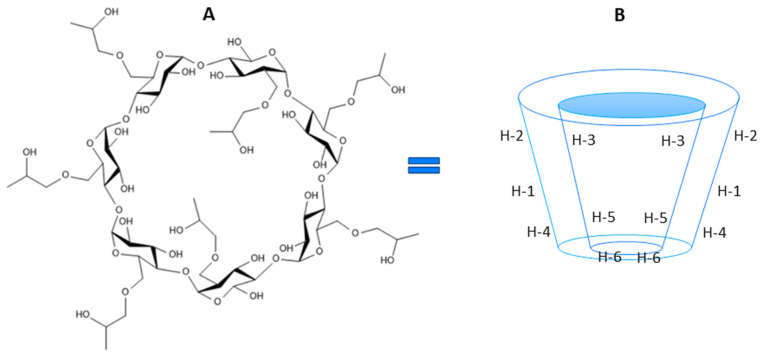
Chemical (**A**) and schematic (**B**) structures of 2-hydroxypropyl-β-CD (HP-β-CD) (based on [34]).

Cyclodextrin derivatives such as 2-hydroxypropyl-β-CD (HP-β-CD) (Figure 2) form highly soluble complexes. HP-β-CD is an alternative to α-, β- and γ-cyclodextrin. In new applications, HP-β-CD is not used as an excipient, but as an active pharmaceutical agent. In 1986, Pitha and co-workers suggested that HP-β-CD administered parenterally as a solubilizer of a poorly soluble drug would not remain inactive after drug release, but could encapsulate lipophilic components, such as vitamins and hormones, in the body [30]. A year later, his group used the intravenous cyclodextrin treatment of HP-β-CD in siblings with hypervitaminosis A (unable to metabolize vitamin A) to remove excess vitamin A. This was the first time HP-β-CD was used as a drug rather than as an adjunct.

These modified forms of β-CD derivatives have gained prominence due to their flexible cavity sizes, greater water solubility, and low toxicity [31]. There are papers that describe the encapsulation of Schiff bases using 2-hydroxypropyl-β-CD [32,33]. In each case, the water solubility increased many times.

Herein, we present a study on the effectiveness of HP-β-CD cyclodextrin for encapsulating hesperetin Schiff bases, an investigation of the physicochemical properties of potential hesperetin derivatives/cyclodextrin systems through different analytical techniques. These included ultraviolet–visible spectroscopy (UV), Fourier transform infrared spectroscopy (FT-IR), scanning electron microscopy (SEM), differential scanning calorimetry (DSC), X-ray diffractometry (XRD), and nuclear magnetic spectroscopy (NMR). Cytotoxic and antibacterial assays were also performed.

The main objective of our work was to increase the bioavailability and biological activity of hesperetin derivatives (HIN, HTSC, and HHSB) by increasing their water solubility. This was expected to be achieved through their complexation with modified cyclodextrin, i.e., the formation of inclusion complexes so-called encapsulation. It is likely that the encapsulation of hesperetin derivatives with cyclodextrin will directly improve the solubility of hesperetin derivatives and indirectly increase the bioavailability and biological activity of these compounds.

## 2. Results and Discussion

### 2.1. Encapsulation of Hesperetin Schiff Bases by HP-β-CD

Cyclodextrin inclusion complexes can be formed during the encapsulation of hydrophobic compounds, and that was the goal of this experiment. The HP-β-cyclodextrin encapsulation of hesperetin and its Schiff bases were prepared through mechanical (mech) and co-evaporation (CV) methods using a molar ratio compound: HP-β-CD = 1:1. The systems formed had the appearance of a white powder, in addition to the HIN kit; in this case, the powder was yellowed. HTSC with cyclodextrin formed a glossy structure.

### 2.2. Identification of Solid Systems of Hesperetin and Its Derivatives with HP-β-CD

#### 2.2.1. Fourier Transform Infrared (FT-IR) Spectroscopy

**Figure 3:**
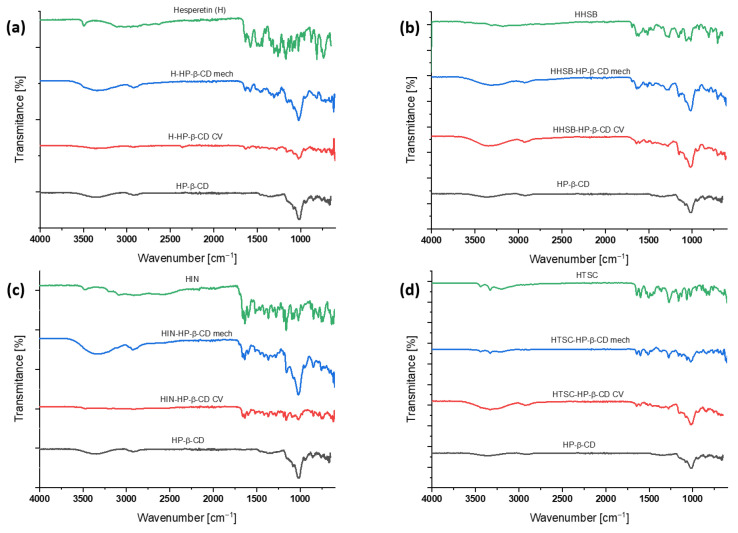
The results of FT-IR analysis of hesperetin (green), physical mixture (blue), the inclusion complex (red), and HP-β-CD (black) (**a**); HHSB (green), physical mixture (blue), the inclusion complex (red), and HP-β-CD (black) (**b**); HIN (green), physical mixture (blue), the inclusion complex (red) and HP-β-CD (black) (**c**); and HTSC (green), physical mixture (blue), the inclusion complex (red), and HP-β-CD (black) (**d**).

FT-IR spectroscopy was applied as a useful technique to provide information about functional groups of studied components. In Figure 3, the infrared spectra of the hesperetin and its derivatives/HP-β-CD systems were analyzed and compared with the spectra of the pure compounds (hesperetin, HHSB, HIN, HTSC, and HP-β-CD).

In Tables S1–S4 (Supplementary Materials), the visible bands on FT-IR spectra for hesperetin, HHSB, HIN, HTSC, and HP-β-CD and their assignments were presented [35,36,37,38,39,40]. The FT-IR spectrum of the physical mixture is the superposition of two compounds. On the other hand, the FT-IR spectra of the H/HP-β-CD (CV) and H/HP-β-CD (mech) revealed no attributes similar to the initial compounds, namely, bands corresponding to the vibration at the B and C rings monitored in hesperetin 2956, 2937, 2876, 2838, 1283, 1093, 1066, 955, and 877 cm^−1^ in spectra of the H/HP-β-CD (CV) and H/HP-β-CD (mech). This image of the spectra suggests the incorporation of the B and C rings of hesperetin into the host cavity—HP-β-CD—which agrees with other authors’ proposals [41].

In the case of hesperetin derivatives (HHSB, HIN, and HTSC), the comparison of the free derivatives with cyclodextrin systems yielded the following results. The most characteristic bands, such as 3614, 3497, 3314, 3181, 3054, 3007, 1455, 1264, 1222, 1067, 1014, 956, and 871 cm^−1^, responsible for vibration at the B and C rings were not observed in HHSB/HP-β-CD (CV) and HHSB/HP-β-CD (mech) systems (Figure 3b). Only a few bands within the B and C rings (1691, 1355, 1292 cm^−1^) were visible in the spectrum of HHSB/HP-β-CD (mech). The peak in the FT-IR spectrum of HP-β-CD at 3362 cm^−1^ became wider in the FT-IR spectrum of the studied systems. This is probably because the vibrations of the HHSB molecule were limited by confining them inside the cyclodextrin molecule. Certain interactions, such as van der Waals force, dipole interaction, or hydrophobic interaction, occurring between host and guest molecules played a crucial role in the formation of the inclusion complex [36].

The spectra of HIN/HP-β-CD and HTSC/HP-β-CD systems (Figure 3c,d) showed physical mixtures. Their spectra are an overlay of HIN or HTSC and HP-β-CD spectra, and no new characteristic absorption peak is generated. 

#### 2.2.2. Powder X-ray Diffraction (PXRD) Studies

Powder X-ray diffraction (PXRD) was applied as helpful technique for determining crystalline and amorphous phases. The results from this analysis can be considered one of the strongest evidences for the formation of an inclusion complex [36].

**Figure 4:**
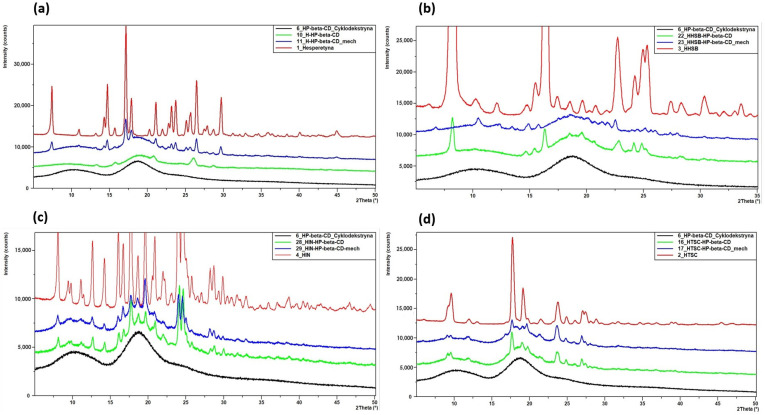
The results of PXRD patterns (viewed from the top) of hesperetin (red), H/HP-β-CD (mech) (blue), H/HP-β-CD (CV) (green), and HP-β-CD (black) (**a**); HHSB (red), HHSB/HP-β-CD (mech) (blue), HHSB/HP-β-CD (CV) (green), and HP-β-CD (black) (**b**); HIN (red), HIN/HP-β-CD (mech) (blue), HIN/HP-β-CD (CV) (green), and HP-β-CD (black) (**c**); and HTSC (red), HTSC/HP-β-CD (mech) (blue), HTSC/HP-β-CD (CV) (green), and HP-β-CD (black) (**d**).

The experiments were carried out at room temperature on the hesperetin and its derivatives with HP-β-CD (Figure 4a–d). The cyclodextrin used in the synthesis is amorphous, which is evident from the broad halo pattern. Pure hesperetin, HHSB, HIN, and HTSC are in the crystalline form, as confirmed by the presence of sharp peaks. 

The PXRD spectra of the physical mixtures obtained are characterized by a superposition of the patterns of the individual constituent compounds, indicating that no new phases are present in the solid state. In the case of complexes, H/HP-β-CD (mech) is a physical mixture of components, while the diffraction pattern of the H/HP-β-CD (CV) sample shows the formation of a new compound with low crystallinity. The low level of crystallinity in the complexes is probably due to the loss of regularity throughout the cross-linked cyclodextrin networks [37]. Moreover, the lack of crystallinity is one of the beneficial proofs for the creation of inclusion complexes. The HHSB/HP-β-CD (mech) sample formed a new phase, while the HHSB/HP-β-CD sample is a physical mixture. On the other hand, both HIN/HP-β-CD and HTSC/HP-β-CD samples, obtained by mechanical and co-evaporation methods, are physical mixtures of components.

#### 2.2.3. Differential Scanning Calorimetry (DSC) Studies

One thermal analysis technique for the qualitative and quantitative analysis of exothermal and endothermic processes is differential scanning calorimetry (DSC).

This allows the determination of crystalline and amorphous forms of various substances and the stability and temperature sensitivity of compounds. The determination of such parameters is very important in the process of product development in the food or pharmaceutical industry [38].

**Figure 5:**
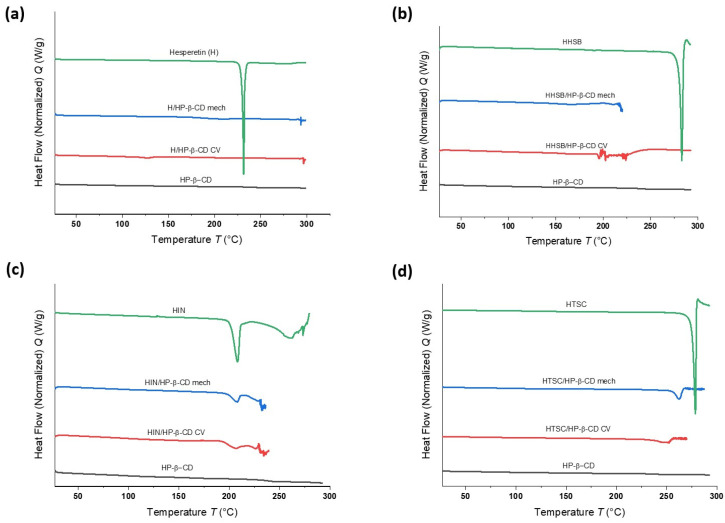
The results of DSC analysis from the second heating scan (viewed from the top) of Hesperetin (green), H/HP-β-CD (mech) (blue), H/HP-β-CD (CV) (red), and HP-β-CD (black) (**a**); HHSB (green), HHSB/HP-β-CD (mech) (blue), HHSB/HP-β-CD (CV) (red), and HP-β-CD (black) (**b**); HIN (green), HIN/HP-β-CD (mech) (blue), HIN/HP-β-CD (CV) (red), and HP-β-CD (black) (**c**); and HTSC (green), HTSC/HP-β-CD (mech) (blue), HTSC/HP-β-CD (CV) (red), and HP-β-CD (black) (**d**).

Two heating cycles were used in each measurement. In the first cycle, the samples were heated from 25 to 100 °C to eliminate the presence of water [35]. The results of the second heating in the 27–290 °C range are shown in Figure 5a–d. On the other hand, the DSC curves recorded for physical mixtures of HHSB/HP-β-CD (mech) (Figure 5b), HIN/HP-β-CD (mech), CV (Figure 5c), and HTSC/HP-β-CD (CV) (Figure 5d) showed the decomposition of the samples at about 220 °C, 233 °C, 231 °C, and 270 °C, respectively.

The DSC curves of hesperetin, HHSB, HIN, and HTSC exhibited sharp endothermic peaks at 231 °C, 283 °C, 208 °C, and 278 °C, respectively, attributed to the melting points of the studied compounds (Figure 5a–d) [18,35]. The peak meaning the endothermic melting process disappears completely in case of cyclodextrin with hesperetin and HHSB, prepared by mechanical and co-evaporation methods, which indicates the loss of their crystallinity, as well as the creation of intramolecular links between the two components of the system. In the case of cyclodextrin systems with HTSC, compared to the initial derivative, a small melting peak appears earlier, at 16 °C and 31 °C for HTSC/HP-β-CD (mech) and HTSC/HP-β-CD (CV), respectively (Figure 5d). For HIN systems with HP-β-CD, the melting peak is visible on each DSC curve (Figure 5c). According to the literature [39], when the deposition of the guest molecule in the cavities of HP-β-CD causes changes in various parameters such as melting, boiling, or sublimation temperatures. The temperature values may shift or disappear completely when the HP-β-CD molecule is decomposed. Hence, the DSC curves in Figure 5a,b,d confirm that fact.

#### 2.2.4. SEM Analysis

**Figure 6:**
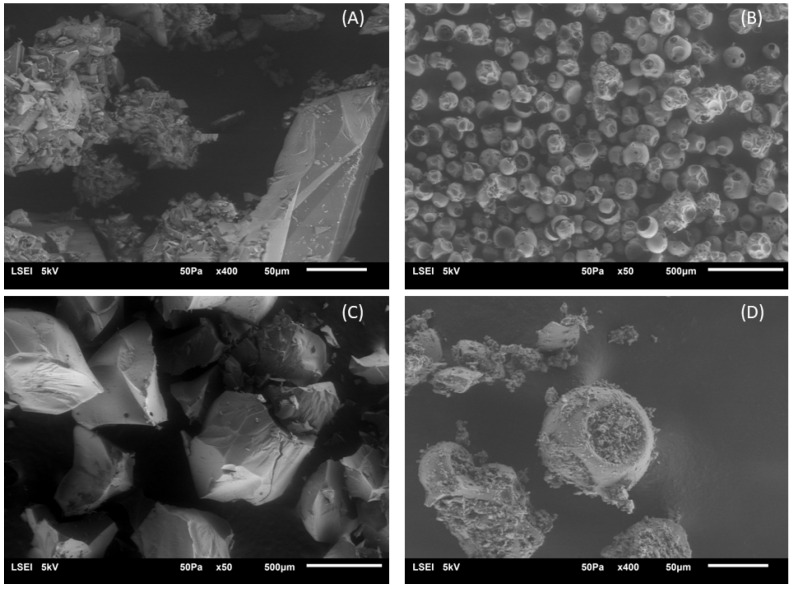
Scanning electron microphotographs: (**A**) hesperetin, (**B**) HP-β-CD, (**C**) hesperetin/HP-β-CD inclusion complex, and (**D**) hesperetin and HP-β-CD physical mixture (1:1 molar ratio).

**Figure 7:**
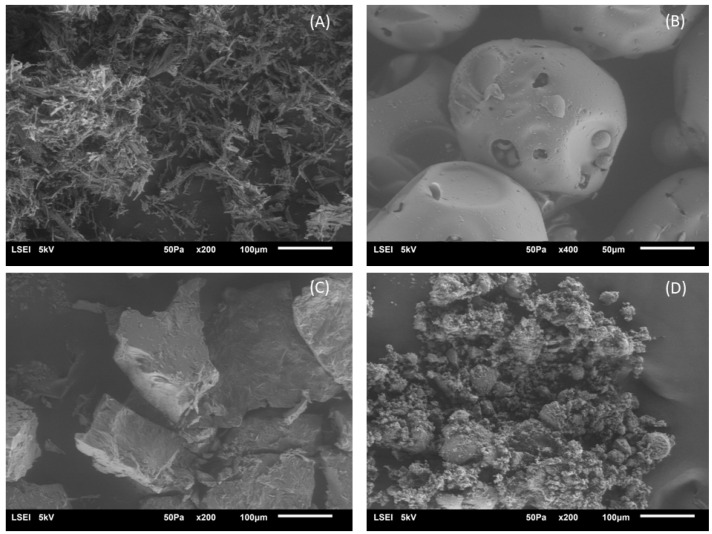
Scanning electron microphotographs: (**A**) HHSB, (**B**) HP-β-CD, (**C**) HHSB and HP-β-CD physical mixture (1:1 molar ratio), and (**D**) HHSB/ HP-β-CD inclusion complex.

To confirm the formation of inclusion complexes by observing their morphology, scanning electron microscopy was used as an auxiliary method [40]. The SEM photographs of HP-β-CD, hesperetin, and Schiff base and their physical mixtures and inclusion complexes are shown in Figure 6 and Figure 7. Typical crystals of HP-β-CD, hesperetin and HHSB (the Schiff base chosen due to the formation of an inclusion complex) are found in many different sizes. Pure hesperetin crystallizes in the form of small platelets, although larger crystalline units also occur (Figure 6A). HP-β-CDs exhibited a spherical shape with cavities (Figure 6B), consistent with the morphology observed in previous reports [41,42]. Some resemblance to the crystals of free molecules was shown by the physical mixture of hesperetin/HP-β-CD (Figure 6D). In contrast, the hesperetin/HP-β-CD inclusion complex was characterized as a compact and homogeneous system with a lamellar structure, and varied considerably from the size and features of hesperetin shapes of HP-β-CD and hesperetin (Figure 6C), which confirms the formation of the hesperetin/HP-β-CD inclusion complex. The combination of substances in the solid state of both hesperetin and HP-β-CD did not form a close compound. The substances still existed in their original forms. In contrast, when the two compounds were combined in solution form, the formation of a close compound/system occurred. Hesperetin no longer existed in it in a crystalline state; therefore, the formation of an inclusion complex is presumed [43].

A similar issue applies to the HHSB/HP-B-CD system (Figure 7). In the physical mixture (Figure 7C), structure is visible as a collection of blocks. There are no similarities to the initial compounds (Figure 7A,B), whereas, the inclusion complex (obtained by mechanical method—Figure 7D) appeared as irregular particles, in which the original morphology of both components almost disappeared, and tiny aggregates of amorphous irregular size pieces were present. In this case, after mixing the solutions of the two compounds, there was no close association, although the structures of the starting substances are not visible in the picture.

### 2.3. Identification of Systems of Hesperetin and Its Derivatives with HP-β-CD in Solutions

#### 2.3.1. Nuclear Magnetic Resonance (NMR) Studies

**Figure 8:**
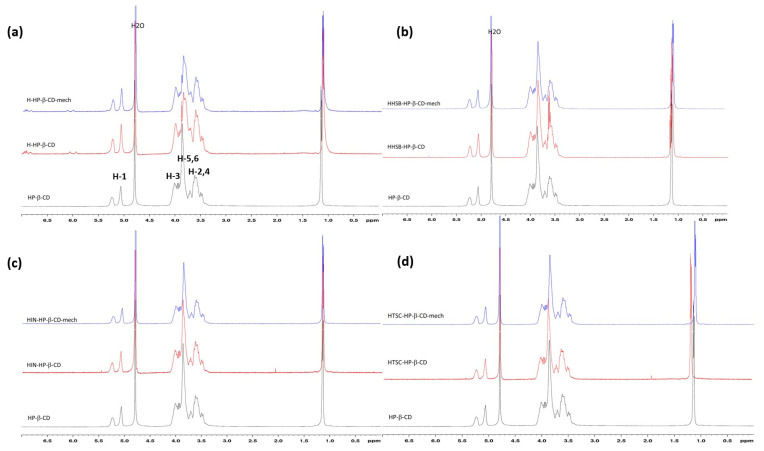
^1^H NMR spectra of hesperetin and its derivatives in the presence of HP-β—CD in D_2_O at 25 °C. (**a**) hesperetin/CD systems, (**b**) HHSB/CD systems, (**c**) HIN/CD systems, and (**d**) HTSC/CD systems.

In order to confirm the interaction between the hesperetin, HHSB, HIN, and HTSC/HP-β-CD complexes, a comparison of the ^1^H NMR spectra in D_2_O solvents of hesperetin and its derivatives in the presence of the host CD (Figure 8) were performed. Due to poor solubility in water, hesperetin is transparent to ^1^H NMR under most conditions, when D_2_O is used as a solvent [44]. 

Six signals for individual hydrogen in the cyclodextrin molecule were identified and characterized. They are as follows: H3 and H5 pointing to the inside of the CD cavity, H1, H2 and H4 located on the outer surface of the CD, and H6 located on the edge of the truncated CD cone. In the process of guest inclusion into the cyclodextrin cavity, the shifts of H3 and H5 of CD should be observed [45]. Due to the presence of glucopyranosyl units in HP-β-CD, which are responsible for the inclusion complex formation, those shifted peaks are undistinguishable. Yang and co-workers [44] confirmed from the ROESY spectrum of the hesperetin/HP-β-CD complex that the C ring of the hesperetin molecule was included in the HP-β-CD cavity.

The evaluation of the hesperetin complex via ^1^H NMR clearly showed the presence of backbone protons of the hesperetin molecule, consistent with significant solubilization. As shown in Figure 8, most of the hesperetin protons showed chemical shifts at δ 5.5–7.5 ppm, which were different from HP-β-CD protons (usually at δ 3.0–5.0 ppm), consistent with the conclusions in reference [44]. In order to accurately analyze the inclusion mode of the studied systems, the chemical shifts of CD protons in the absence and presence of hesperetin and its derivatives were examined (Table 1). Inclusion complexation with hesperetin had a significant effect on the δ values of H-5 HP-β-CD protons (in the range of 0.0371–0.0328 ppm), and a negligible effect on the other compounds (0.0037–0.010 ppm). In contrast to this, the values of protons H-2, H-3, H-4, and H-6 showed relatively weak changes (0.0001–0.0145 ppm). Interestingly, among the tested compounds for the aforementioned four CD protons, the largest, although very insignificant, changes were characterized by the system with HTSC obtained by co-evaporation (H3–H6: 0.01–0.0145 ppm). It could have been caused by the presence of a nitrogen tail of HTSC located inside the cavity of the CD and close to the primary -OH [46].

In all studied cases, except for HTSC, the shifts for H-3 and H-5 protons are negligible. Although for the two systems H/HP-β-CD (CV) and HHSB/HP- β -CD (mech) methods such as FT-IR, PXRD, or DSC confirmed the formation of an inclusion complex, shifts for H-3 and H-5 protons were not observed. Ficarra and co-workers [47] observed a similar phenomenon analyzing systems between β-cyclodextrin and flavonoids, including hesperetin. In their case, the ^1^H-NMR spectra showed up-field shifts, due to the diamagnetic anisotropy of the attached guest of the H-3 and H-5 proton signals combined with slight shifts of the H-6 signal, while the H-1, H-2, and H-4 signals located outside the cavity are relatively unchanged; these results indicated an interaction of part of the molecule with the cavity -CD, and consequently the formation of an inclusion complex between each flavonoid and CD. 

The order of the up-field shift for H/HP-β-CD (mech) was found to be H-5 (∆δ/ppm = 0.0371) > H-1 (0.0035) > H-4 (0.0029) > H-6 (0.0023) > H-3 (0.0012) > H-2 (0.0005); for H-HP-β-CD (CV) it was H-5 (∆δ/ppm = 0.0328) > H-6 (0.0053) > H-3 (0.0049) > H-1 (0.0021) > H-4 (0.0017) > H-2 (0.0004); for HHSB/HP-β-CD (mech) it was H-3 (∆δ/ppm = 0.0077) > H-5 (0.0075) > H-6 (0.0075) > H-1 (0.0032) > H-4 (0.0026) > H-2 (0.0001); for HHSB/HP-β-CD (CV) it was H-4 ((∆δ/ppm = 0.028) > H-5, H-6 (0.0069) > H-3 (0.0059) > H-1 (0.0027) > H-2 (0.0012); for HTSC/HP-β-CD (mech) it was H-4 (∆δ/ppm 0.0042) > H-5, H-6, H-3, H-1 (0.0037) > H-2 (0.0035); for HTSC/HP-β-CD (CV) it was H-4 (∆δ/ppm 0.0145) > H-3 (0.011) > H-5, H-6, H-Me (0.0103) > H-1 (0.0102) > H-2 (0.0056); for HIN/HP-β-CD (mech) it was H-5, H-6 (∆δ/ppm 0.0068) > H-1 (0.0065) > H-2 (0.0061) > H-3 (0.0060) > H-4 (0.0054); for HIN/HP-β-CD (CV) it was H-2 ((∆δ/ppm 0.0106) > H-1 (0.0103) > H-4 (0.0084) > H-6, H-5, H-3 (0.0079). It was observed that in some cases, the shifts in protons located outside the torus (H1, H2, and H4) were relatively larger than for H3 and H5 inside the capsule.

#### 2.3.2. UV-Vis Spectroscopy Studies

**Figure 9:**
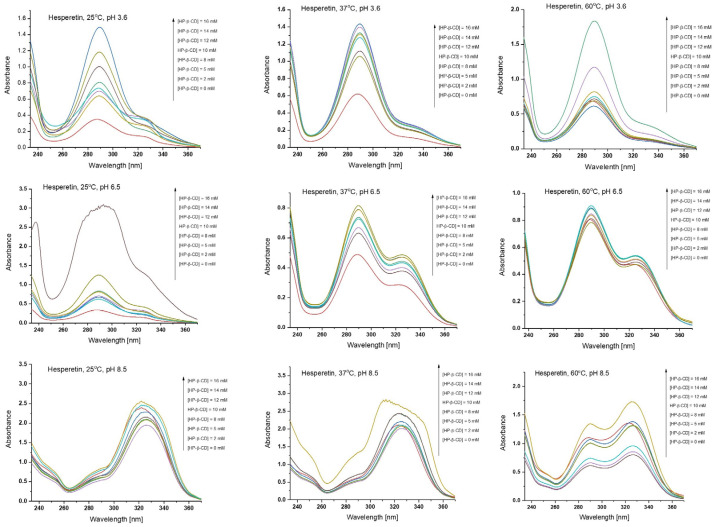
UV absorption spectra of hesperetin in the presence of increasing concentrations of HP-β-CD (0.0–16 mM) at various pH and temperatures.

The effect of HP-β-CD on the UV absorption spectra of the tested materials was quantitatively investigated in three different media, namely acetate buffer (pH 3.6), phosphate buffer (pH 6.5), and borate buffer (pH 8.5), and temperatures (25, 37, and 60 °C). An excess amount of each the guest was added to the host concentration between 0.0 and 16 mM. At this concentration range, the absorbance value was significantly high, as can be seen in Figure 9, hence the choice of max concentration at 16 mM. Moreover, studies in this concentration range were also presented by Tommasini et al. [48]. The concentration values of the tested substances were determined by UV-Vis spectrophotometry. According to the literature [48] the absorption maxima were progressively shifted to longer wavelengths, especially during the move to increasingly higher pH values of buffers (from 287 to 326 nm), as shown by the example of hesperetin (Figure 9). Moreover, at pH 6.5 and 8.5, bands at 287 and 322 nm, respectively, could be seen. UV absorption spectra of flavonoids are characterized by the presence of two main absorption bands. Band I (in the 300–400 nm range) and band II (in the 240–300 nm range) respond to the absorption of the cinnamoyl system and the benzoyl grouping in the molecules, respectively. Due to the weak acidity of phenolic hydroxyl groups in flavonoids, the aforementioned absorption bands are very sensitive to the effect of pH. An increase in the intensity of shifted band II (323 nm) is observed with increasing pH. The absorbance of the shifted II band was indirectly due to the degree of ionization of the phenolic hydroxyl group. For pH 6.5, the moderate intensiveness of the shifted II band reflected the coexistence of both neutral and anionic moieties. At pH 8.5, there was complete dissociation of the phenolic -OH group [48].

#### 2.3.3. Phase Solubility Studies

The purpose of this part of the study was to determine the solubility of compounds of hesperetin and its Schiff bases (HHSB, HIN, and HTSC) in aqueous solution at different pHs and temperatures, and the effect of their inclusion in modified HP-β-CD on the aqueous solubility of these compounds at different pH values.

The effect of the presence of HP-β-CD at different temperatures (25, 37, and 60 °C) was determined by developing phase solubility diagrams. The plots of solubilized compounds against the cyclodextrin concentration showed good linearity. The results of the research are presented in Table 2. 

In accordance with the methodology described in Section 3.4, excess amounts of each tested substance were added to aqueous solution with increasing concentration of HP-β-CD up to 16 mM, respectively, in 2 mL of 100 mM sodium acetate buffer (pH 3.6), 100 mM sodium phosphate buffer (pH 6.5) or 100 mM sodium borate buffer (pH 8.5). At pH 3.6 for samples of hesperetin, HHSB, HIN, and HTSC with HP-β-CD, the maximum absorbances were recorded at wavelengths of 287, 320, 321, and 323 nm, respectively. At pH 6.5—hesperetin (289 nm), HHSB (227 nm), HIN (381 nm), and HTSC (324 nm). In alkaline buffer—hesperetin (324 nm), HHSB (227 nm), HIN (389 nm), and HTSC (324 nm). A tendency for the UV bands to shift toward higher wavelengths depending on the environment was observed.

**Figure 10:**
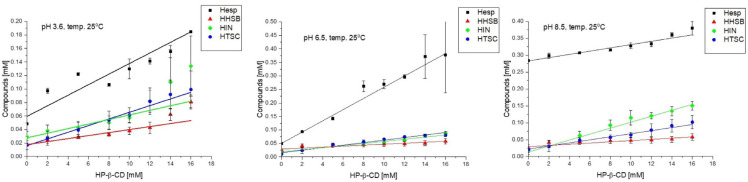
Phase of solubility diagram of hesperetin and its Schiff bases (HHSB, HIN, and HTSC) in sodium phosphate buffer (pH 6.5) with HP-β-CD.

According to Figure 10, it can be noted that compounds’ concentration increased linearly with HP-β-CD concentration. At 25 °C, hesperetin solubility with HP-β-CD at 16 mM increased the most at pH 6.5 (five-fold) (Table 2). Lucas-Abellán et al. [49] presented the increase hesperetin solubility in this medium by 164 times, but the highest concentration of cyclodextrin in their assay was 100 mM. At room temperature, in the presence of HP-β-CD HTSC hesperetin dissolved moderately; its highest dissolution rate (five-fold) can be seen at pH 6.5. At the same temperature, an increasing trend in solubility (from 0.017 ± 0.006 to 0.09 ± 0.05) with increasing pH was observed for HIN. In the case of HHSB, changes in solvent multiplicity with an increase in pH at room temperature were not observed.

At 37 °C (a temperature close to that of the human body), the largest changes in solubility were observed for HHSB and HIN. At pH 6.5, solubility for HHSB and HIN increased by 8 and 15 times, respectively.

At higher temperatures, i.e., 60 °C, no significant effect of cyclodextrin was recorded for the solubility of the tested compounds. Relative to hesperetin, this increased ability in solubility was noted for its derivatives, such as HTSC and HIN at pH 3.6 (five-fold), HHSB and HIN at pH 6.5 (four- and five-fold, respectively), and HHSB at pH 8.5 ((five-fold).

The phase solubility plots for the compounds-HP-β-CD complexes displayed typical A_L_ diagrams, meaning that compound solubility increases linearly with increasing HP-β-CD concentration (Figure 10). The slope values lower than 1 indicates a 1:1 stoichiometry between host and guest [49].

The complexation constant (K_c_) values obtained for hesperetin were lower in comparison to literature reports [49,50]. Nevertheless, the dependence of the values on the pH of the environment is preserved, i.e., K_c_ values are highest at pH 6.5 and lowest in an alkaline environment. Analyzing the values of K_c_ (from the highest values), it can be observed that the complexes were formed between HP-β-CD and hesperetin, HHSB, HIN, and HTSC. It is known that all flavonoids deprotonate at the C7 position in an alkaline pH (8.5) environment. In addition, in a pH 7.5–8.0 environment, flavanones tend to form chalcone. Thus, in a more alkaline environment (pH 8.5), due to the high water solubility of the chalcone form, the value of the complexation constant drops significantly [50].

Generally, the K_c_ values represent the strength of the interaction between guest and host, providing the opportunity to estimate the affinity between all tested compounds. Therefore, in order to study in more depth the effect of CD solubility on hesperetin derivatives, complexation efficiency (CE) values were calculated. The CE represents the molar ratio between complexed and the free CD concentration [51]. Regarding both the water solubility of flavanone and Kc values for complexes with 1:1 stoichiometry, the CE parameter was calculated based on the slope of the phase solubility diagram using Equation (2). The CE values obtained for all studied compounds with HP-β-CD are shown in Table 2. As can be seen in Table 2, the highest values of this parameter were assigned to hesperetin under all conditions tested in this work.

The CE values were also managed to determine the molar ratio in solution of Schiff bases:CD or hesperetin:CD, the value of which would reflect the expected increase in tested compounds’ solubility with HP-β-CD (Equation (3)). The values of the molar ratio obtained for all tested compounds with HP-β-CDs are shown in Table 2. Among all compounds tested, only pure hesperetin had a low molar ratio, followed in order by HHSB. For example, at pH 6.5 at 25 °C, one molecule of cyclodextrin in solution formed water-soluble complexes with hesperetin. The highest molar ratio (1:30) for hesperetin was recorded at pH 3.6 at 25 °C. For HHSB, the highest molar ratio (1:668) was determined at pH 8.5 at 37 °C. HIN and HTSC molecules require much larger amounts of cyclodextrin molecules to form a water-soluble complex (Table 2). This fact may explain the difficulties in obtaining inclusion complexes of these compounds

### 2.4. Antioxidant Activity Analysis

To evaluate the antioxidant ability of the tested systems, antioxidant analysis was performed. Antioxidants can scavenge hydroxyl radicals in two ways: by reacting directly with hydroxyl radicals (DPPH^•^, ABTS^•+^) or indirectly inhibiting the formation of hydroxyl radicals by chelating metal ions involved in the production of hydroxyl radicals [52,53]. The antioxidant activity of the systems studied was investigated by using DPPH^•^ scavenging, ABTS^•+^ scavenging ability, and Fe^2+-^chelating ability. In this study, the antioxidant activity of pure hesperetin and its Schiff bases (HHSB, HIN, and HTSC) and HP-β-CD complexes were compared, and the positive control was Trolox.

**Figure 11:**
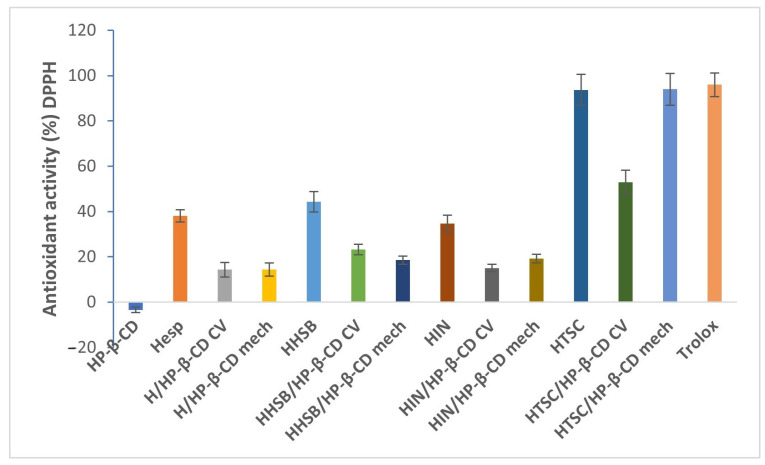
Antioxidant activity comparison by DPPH assay between hesperetin, HHSB, HIN, HTSC, and CD complexes at a concentration of 0.1 mg/mL; values represented as mean ± SD.

As shown in Figure 11, the antioxidant activity of all the examined samples has been found to be lower than that of Trolox. Compared with pure hesperetin (38.08 ± 2.7%) and its Schiff bases (HHSB—44.29 ± 4.5%, HIN—34.62 ± 3.8%, HTSC—93.72 ± 6.8%) the DPPH antioxidant activity decreases in the presence of HP-β-CD, except for one case of HTSC/HP- β-CD (mech), for which DPPH antioxidant activity was 93.97 ± 7.0%.

It was found that the concentration of the host or guest material and form of guest material influence antioxidant activity [54]. In other words, DPPH radical scavenging activity was increasing depending on guest concentration, and it was strongly observed for cyclodextrin complexes. Presumably, the concentration of cyclodextrin used in complexes with Schiff bases was too low to show enhanced scavenging of the DPPH radical. The studied complexes were produced with a small number of pure ligands through a molecular inclusion process, and thus most of the ligands were probably not incorporated into HP-β-CD. A negligible number of pure ligands/guests was slightly miscible in water without a water-soluble carrier, but over a certain concentration, pure compounds could not dissolved entirely. It was observed that the solubility of ligands was improved by forming HP-β-CD complexes. This fact was confirmed by Wdowiak et al. [35] in the DPPH antioxidant activity assay; the enhanced antioxidant assay was observed for Het/HP-β-CD in a 1:2 molar ratio. As a reminder, in the present work the molar ratio for all systems (ligand/HP-β-CD) was 1:1.

**Figure 12:**
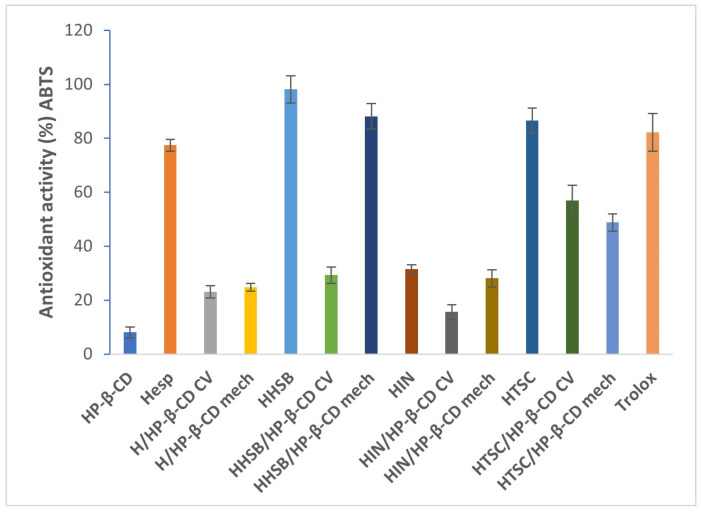
Antioxidant activity comparison using ABTS assay between hesperetin, HHSB, HIN, HTSC, and CD complexes at a concentration of 0.1 mg/mL; values represented as mean ± SD.

In Figure 12, the results of ABTS^•+^ assay showed similar trend, with the DPPH assay of tested HP-β-CD complexes, while hesperetin (77.42 ± 2.2%) and its Schiff bases (HHSB—98.11 ± 5.0%, HIN—31.56 ± 1.6%, HTSC—86.61 ± 4.6%) showed higher antioxidant activity than the complexes. In this case, the HHSB/HP-β-CD (mech) complex showed the highest value of antioxidant activity (88.15 ± 4.7%) among the analyzed complexes.

**Figure 13:**
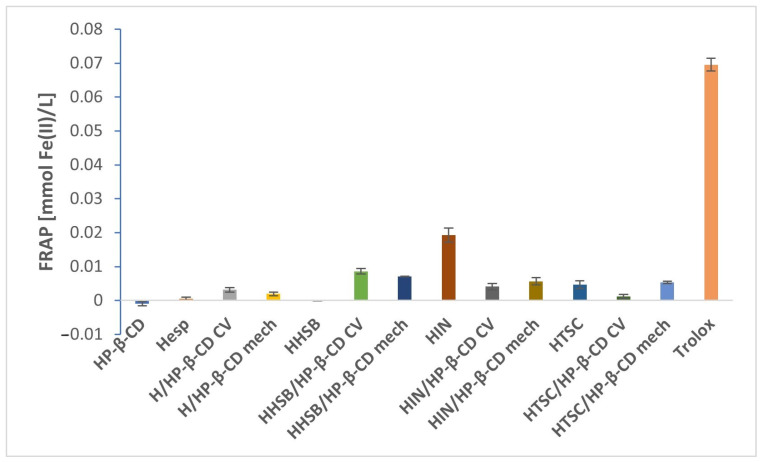
Antioxidant activity comparison of hesperetin, HHSB, HIN, HTSC, and CD complexes using FRAP assay; values represented as mean ± SD.

The results of the FRAP assay (Figure 13) presented a slightly modified trend compared to previous tests. All hesperetin and HHSB complexes and HTSC/HP-β-CD (mech) showed higher values of antioxidant activity than pure compounds. The activity values of the individual complexes are as follows: H/HP-β-CD (0.003 ± 0.0007 mmolFe(II)/L), H/HP-β-CD (mech) (0.002 ± 0.0005 mmolFe(II)/L), HHSB/HP-β-CD (0.009 ± 0.0008 mmolFe(II)/L), HHSB/HP-β-CD (mech) (0.007 ± 0.00008 mmolFe(II)/L) and HTSC/HP-β-CD (mech) (0.005 ± 0.0003 mmolFe(II)/L) versus hesperetin (0.0005 ± 0.0005 mmolFe(II)/L), HHSB (0.000005 ± 0.00005 mmolFe(II)/L), HTSC (0.000005 ± 0.00005 mmolFe(II)/L). An explanation of this phenomenon may be that in FRAP method the reduction of ferric ions is performed using protons and electron donor groups such as the hydroxyl group (hesperetin and its derivatives possess three such groups of this type) and thus in combination with HP-β-CD, the ability to release electrons and protons is improved, as evidenced by the increase in the reducing capacity compared to the basic compounds [20]. Regarding the rest of the systems, these results may show that HP-β-CD is simply mixed with HIN and HTSC, not forming any kind of complex with it. Therefore, HIN/HP-β-CD and HTSC/HP-β-CD (CV) are less capable of chelating of metal ions than the previously mentioned complexes.

### 2.5. Cytotoxic Activity of Tested Chemicals

**Figure 14:**
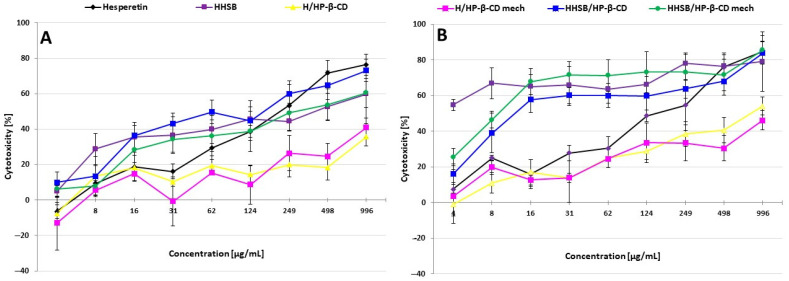
Cytotoxicity of tested chemicals after (**A**) 24 and (**B**) 48 h of exposure of HaCaT cells (human keratinocyte) to MTT (3-(4,5-Dimethylthiazol-2-yl)-2,5-Diphenyltetrazolium Bromide) assay. Each point represents the mean absorbance values of the four replicates from three independent experiments (±standard deviation of the mean—S.E.M.).

HaCaT keratinocyte cells were exposed to chemical compounds at nine different concentrations (from 3.9 to 995.6 µg/mL) in four replicates of each concentration for 24 and 48 h. The curves of the dependence of cytotoxicity on test concentration for selected samples are presented in Figure 14A,B and show results from three independent experiments as mean ± standard deviation of the mean (S.E.M). The rest of the samples are included in Figure S1 in Supplementary Materials. In the presence of some compounds tested at the lowest concentration (4 µg/mL), stimulated proliferation and viability of HaCaT cells was observed (Figure S1A,B). This phenomenon is essentially a rescue mechanism, as cells avoid detrimental stimuli through the induction of proliferation [55].

The curves obtained after 24 h of exposure showed that for most of tested compounds, cytotoxicity increased with the concentration of the chemicals (Figure 14A). The only exception was the HP-β-CD compound, for which cytotoxicity remained consistently low (up to 6.6% ± 7.2%) (Figure S1A). The compound H/HP-β-CD also induced weak cytotoxicity, up to 35.7% ± 5.1% at the highest tested concentration (995.6 µg/mL). Its H/HP-β-CD (mech) derivative showed slightly higher but similar cytotoxicity, up to 40.9% ± 5.5% in the presence of the highest tested concentration. In contrast, hesperetin and the compound HHSB/HP-β-CD proved to be the most cytotoxic. These results are correlated with achieved IC_50_ values (Table 3). The IC_50_ values for hesperetin and the compound HHSB/HP-β-CD were 220.9 µg/mL and 166.7 µg/mL, respectively. According to the calculation of the IC_50_ values, the degree of cytotoxicity of the tested compounds was as follows (from the most to the least cytotoxic): HHSB/HP-β-CD > hesperetin > HHSB/HP-β-CD (mech) > HIN > HTSC/HP-β-CD (mech) > HIN/HP-β-CD (mech) > HHSB > HTSC/HP-β-CD > HIN/HP-β-CD. For HTSC, HP-β-CD, H/HP-β-CD, and H/HP-β-CD (mech), no IC_50_ was determined after 24 h of exposure due to too low cytotoxicity. Non-cytotoxic concentrations (IC_0_) ranked as follows (from highest to lowest): HP-β-CD and H/HP-β-CD (mech) ≤ 31.1 µg/mL; HIN/HP-β-CD and HIN/HP-β-CDmech ≤ 15.6 µg/mL; HIN ≤ 7.8 µg/mL; and remaining compounds ≤3.9 µg/mL.

After 48 h of exposure, cytotoxicity increased markedly for all compounds tested. An analysis of the obtained curves shows again that observed cytotoxicity was positively correlated with the test concentration (Figure 14 and Figure S1A,B), with the exception of the HHSB compound, for which cytotoxicity was very high and at the lowest concentration tested (3.9 µg/mL) was 54.8% ± 5.4, while at the highest (995.6 µg/mL) was 79.1% ± 6.6. HHSB/HP-β-CD, HHSB/HP-β-CD (mech), and HTSC showed comparable high cytotoxicity. The achieved results are correlated with IC_50_ values (Table 3). HHSB was the most cytotoxic, with IC_50_ impossible to count, so IC_70_ was calculated, which was 163.5 µg/mL. According to the calculation of the IC_50_ values, the degree of cytotoxicity of the tested compounds after 48 h exposure was as follows (from the most to the least cytotoxic): HHSB/HP-β-CD (mech) > HTSC > HHSB/HP-β-CD > HIN > hesperetin > HTSC/HP-β-CD > HTSC/HP-β-CD (mech) > HIN/HP-β-CD (mech) > HIN/HP-β-CD > H/HP-β-CD. H/HP-β-CD (mech) showed comparable cytotoxicity after both exposure times, and due to its too-low cytotoxicity, no IC_50_ value was determined for this compound, as with HP-β-CD. Non-cytotoxic concentrations (IC_0_) ranked as follows (from highest to lowest): HIN ≤ 15.6 µg/mL; HIN/HP-β-CD ≤ 7.8 µg/mL; hesperetin, HTSC, HP-β-CD, H/HP-β-CD, H/HP-β-CD (mech), HTSC/HP-β-CD, HTSC/HP-β-CD (mech), and HIN/HP-β-CD (mech) ≤ 3.9 µg/mL. For HHSB, HHSB/HP-β-CD, and HHSB/HP-β-CD (mech), non-cytotoxic concentration was not determined because the cytotoxicity of the lowest test concentration was slight or mild.

### 2.6. Antibacterial Activity of Tested Chemicals

In vitro antibacterial screening of hesperetin, its Schiff bases, and complexes with modified cyclodextrin was assayed against various pathogenic bacterial strains. The antimicrobial activity results of hesperetin, its Schiff bases, and complexes with modified cyclodextrin are presented in Table 4. We found that both the Schiff bases and hesperetin’s cyclodextrin complexes exhibited variable antimicrobial activity depending on the type of bacterial strain.

The tested compounds showed variable activity against two Gram-positive (*Staphylococcus aureus* ATCC 25923, *Staphylococcus aureus* ATCC 29737) and two Gram-negative (*Escherichia coli* ATCC 11303 and *Escherichia coli* ATCC 35218) bacteria (Table 4 and Table 5). The hesperetin itself effectively inhibited the bacterial growth, showing MIC values of 2.5 µM against *Escherichia coli* ATCC 11303, and its activity was comparable to that for cyclodextrin complexes. The literature indicated that hesperetin possess antimicrobial activity [56]. According to published results [1] hesperetin may exhibit a higher antibacterial effect on Gram-positive bacteria than on Gram-negative bacteria. Hesperetin effectively inhibited the bacterial growth showing MIC values at level of 125 µg/mL against *S. aureus* and 500 µg/mL against *E. coli* [NO_PRINTED_FORM]. Compared to the present study, the values were significantly smaller (50000 fold). There have been extensive studies about the protective effect of hesperetin against toxicity caused by microorganisms and some chemotherapy drugs [57,58]. The precise mechanisms of their antimicrobial activity are not fully understood, but various pathways have been suggested, such as causing cell membrane damage, inhibiting various synthases that participate in the synthesis of nucleic acids, the bacterial respiratory chain, or cell envelope synthesis [59,60]. Nevertheless, a number of works suggest that the increased lipophilicity of flavonoids may enhance antimicrobial activity, especially among aglycones [54,55,59,61].

Regarding MIC values (Table 5), HTSC showed antimicrobial activity only against Gram-positive bacteria at level 2.5 and 1.25 µM. HHSB and HIN acted at 2.5 µM against the *Staphylococcus aureus* ATCC 25923, *Escherichia coli* ATCC 35218 (HHSB), and *Escherichia coli* ATCC 11303 (HIN). An MIC value of 2.5 µM for HP-β-CD did not occur only in case of *Escherichia coli* ATCC 35218.

All complexes with modified cyclodextrin inhibited *Escherichia coli* ATCC 11303 and *Staphylococcus aureus* ATCC 25923. *Escherichia coli* ATCC 35218 was suppressed only by HTSC/HP-β-CD (mech) while *S. aureus* ATCC 29737 was susceptible to the action of the complexes obtained by the co-evaporation method.

The lipophilicity of flavonoids is an important factor for antimicrobial activity, but the studies for the tested systems have not been carried out. However, according to the literature, sometimes high lipophilicity values are not related to the high antimicrobial activity in many cases [61,62,63]. In this study, 5% of DMSO did not sufficiently increase the solubility of hesperetin and its Schiff bases, and there were some precipitates in the bacterial culture broth at high concentrations of the tested compounds. The higher antimicrobial activity of cyclodextrin complexes can be attributed to cyclodextrin’s solubility being higher than hesperetin and its modifications.

To sumarize the MIC values, significantly enhanced antimicrobial activity for the obtained complexes was not observed.

## 3. Materials and Methods

### 3.1. Materials

The racemic hesperetin, 2,2-Diphenyl-1-picrylhydrazyl (DPPH), 2,2′-Azino-bis(3-ethylbenzothiazoline-6-sulfonic acid) diammonium salt (ABTS), (±)-6-Hydroxy-2,5,7,8-tetramethylchromane-2-carboxylic acid (Trolox), potassium persulfate, glacial acetic acid, and hydrochloric acid were purchased from Sigma-Aldrich Co. (Poznań, Poland). Ethanol anhydrous 99.8%, pure for basic analysis, was purchased from POCH (Gliwice, Poland). Sodium acetate anhydrous and ferric chloride hexahydrate were purchased from EUROCHEM BGD (Tarnów, Poland). Ferrous sulphate heptahydrate was purchased from CHEMPUR (Piekary Śląskie, Poland). (2-Hydroxypropyl)-β-cyclodextrin (HP-β-CD, average M.W. 1505 [M + Na^+^] g/mol, Purity: 99% 19.6–26.3% (-OCH2CHOHCH3), Degree of Substitution (DS) 4.9) was purchased from AmBeed (Arlington Hts, IL, USA), CAS No.: 128446-35-5, Cat. No.: A405837. 2,4,6-Tripyridyl-S-triazine (TPTZ) was purchased from Fluorochem Limited (Hadfield, UK). Hesperetin derivatives HHSB (*N*-[2,3-dihydro-5,7-dihydroxy-2-(3-hydroxy-4-methoxyphenyl)chromen-4-ylidene]isonicotinohydrazide), HIN (*N*-[2,3-dihydro-5,7-dihydroxy-2-(3-hydroxy-4-methoxyphenyl)chromen-4-ylidene]benzhydrazide) and HTSC (*N*-[2,3-dihydro-5,7-dihydroxy-2-(3-hydroxy-4-methoxyphenyl)chromen-4-ylidene]thiosemicarbazide) are characterized in references [10,11]. All reagents were of analytical quality and were used without further purification.

### 3.2. Encapsulation in the Solution

Encapsulation of hesperetin and its derivatives (HHSB, HIN, and HTSC) with HP-β-CD were prepared in molar ratio 1:1 via co-evaporation (CV) or co-precipitation method. To a stirring ethanolic solution of hesperetin and its derivatives at room temperature, a clear saturated aqueous solution of HP-β-CD was added dropwise. The mixture was stirred for 24 h at 30 °C, then the temperature was decreased at room temperature, and was continued until the solvent was evaporated. The homogeneous paste was dried at 40 °C until it reached a constant weight [20]. The completed samples were stored in a desiccator with a drying agent.

### 3.3. Preparation of Physical Mixture

The physical mixture was prepared by mixing HP-β-CD and hesperetin or its derivatives (HHSB, HIN, and HTSC) in the same molar ration as that used to prepare inclusion complex. Hesperetin or its derivatives (HHSB, HIN, and HTSC) were ground with HP-β-CD for 15 min to ensure homogeneous blend [36]. The completed samples were stored in a desiccator with a drying agent.

### 3.4. Phase Solubility Studies

Phase solubility studies were conducted according to [64] with some modifications. Excess amounts of each tested substance were added to aqueous solution with increasing concentration of HP-β-CD up to 16 mM, in 2 mL of 100 mM sodium acetate buffer (pH 3.6), 100 mM sodium phosphate buffer (pH 6.5), or 100 mM sodium borate buffer (pH 8.5), respectively. The samples were maintained in an ultrasonic bath at various temperatures (25, 37, and 60 °C) for 60 min to reach equilibrium. The aqueous solutions were then filtered through 0.45 µm acetate cellulose (CA) membrane filter and diluted. The tested compound concentration on each clear filtrate was spectrophotometrically determined by measuring at their wavelength maximum [29].

The complexation constant (K_c_) between studied molecules and HP-β-CD was determined by using Equation (1) [64]:

(1) $$K_{c}=\frac{\mathrm{slope}}{S_{0\;}\left(1-\mathrm{slope}\right)}$$

where S_0_ means the water solubility of the studied substance, and “slope” means the slope of the phase solubility diagram.

Complexation efficiency was calculated from the slope of the phase solubility diagram using Equation (2) [65]:

(2) $$\mathrm{CE}=S_{0}\times K_{c}=\frac{\mathrm{slope}}{1-\mathrm{slope}}$$

The CE was also applied to evaluate the molar ratio in solution of tested systems using Equation (3) [51]:

(3) $$\mathrm{Compound}:\mathrm{HP}-\beta -\mathrm{CD}=1:\left(1+\frac{1}{\mathrm{CE}}\right)$$

### 3.5. Physico-Chemical Inclusion Compounds’ Characterization

UV-Vis spectra of the compounds in buffers were recorded in the λ interval 200–900 nm using a Hewlett-Packard 8453 spectrophotometer running 845× UV-Visible ChemStation Software (Agilent, Mulgrave, VIC, Australia). Solutions were inserted in a quartz cell with a path length of 1 cm.

Fourier transform infrared spectroscopy (FTIR) spectra of hesperetin and its derivatives, HP-β-CD and their inclusion compounds were obtained using and analyzed using an FT-IR spectrometer Nicolet 6700 (Thermo Scientific, Waltham, MA, USA) in the range of 350–4000 cm^−1^. The FT-IR spectra of the inclusion complexes were compared with those of pure hesperetin, HP-β-CD and physical mixture.

NMR spectra (^1^H) were obtained at Avance Neo 400 MHz spectrometer (Bruker, Germany) in D_2_O (Deutero Gmbh, Kastellaun, Denmark). Chemical shifts were referenced to the solvent (^1^H: 4.79 ppm for D_2_O) [66] and were expressed in parts per million (ppm, δ).

Thermal analysis was performed using a TA Discovery DSC 2500 (New Castle, DE, USA). Samples weighing about 1.2–2.5 mg were contained in corrugated aluminum pans with a small hole in the lid. Two heating cycles were used. First cycle: heating to 100 °C and holding this temperature for 5 min to remove water; second cycle: samples were cooled to 25 °C and reheated to 300 °C. The constant heating rate of the measurement was 10° K·min^−1^ in a nitrogen atmosphere with a flow rate of 10 mL·min^−1^. 

The detailed methodology of powder X-ray diffraction (PXRD) was described in the previously paper by Sykuła et al. [19].

The structure and morphology of the samples were examined using JEOL JSM-6610LV scanning electron microscope (JEOL Ltd., Tokyo, Japan) working in low vacuum mode.

### 3.6. Antioxidant Activities Research in Vitro

#### 3.6.1. DPPH Assay

The detailed methodology of DPPH assay was described in the previous paper by Sykuła et al. [18].

#### 3.6.2. ABTS^•+^ Radical Cation Decolorization Assay

Antioxidant activity using ABTS^•+^ cation radical scavenging was determined according to a modified method [67]. ABTS solution (2.5 mM) was mixed with 1.0 mM potassium persulfate solution and left at room temperature in the dark for 12-24 h. After this time, the absorbance of the finished ABTS^•+^ solution was corrected with ethanol to 0.70 ± 0.02 at 745 nm. A total of 100 µL of the sample was reacted for 5 min with 2 mL of ABTS^•+^ solution (A_745_ nm = 0.70 ± 0.02). The absorbance value of the reaction mixture was measured at 745 nm. The percentage antioxidant activity was related to the standard Trolox solution as a positive control, and was calculated using the following equation [54]

Scavenging activity (%) = [1 − (A_sample_ − A_blank_)/A_control_] × 100% (4)

where A_control_ is the absorbance value of the control (ABTS^•+^ solution with water), A_blank_ is the absorbance value of samples mixed with anhydrous ethanol, and A_sample_ is the absorbance value of the added test sample to ABTS^•+^ solution at 745 nm, respectively.

#### 3.6.3. Ferric-Ion Reducing Antioxidant Power

The antioxidant capacity determined by the FRAP assay (the ability of plasma to reduce iron) was carried out using a modification of the FRAP assay [67]. The FRAP reagent consisted of the following solutions: 300 mM acetate, pH 3.6 glacial acetic acid buffer, 20 mM ferric chloride (FeCl_3_×6H_2_O), and 10 mM 2,4,6-tripyridyl-S-triazine (TPTZ) prepared in 40 mM HCl. The aforementioned solutions were mixed together in a ratio of 10:1:1 (*v*/*v*/*v*). The FRAP assay consisted of adding 15 µL of sample to 1.98 mL of reagent and incubating the mixture at 37 °C for 4 min. Absorbance measurement was recorded at 593 nm against a blank. Total antioxidant capacity was estimated relative to a standard of known FRAP concentration, ferrous sulfate. The percent of antioxidant activity was compared to a standard solution of Trolox as a positive control.

### 3.7. Cytotoxic Activity

#### 3.7.1. Preparation of Test Chemicals

All compounds were dissolved in sterile DMSO (Merck Life Science, Warsaw, Poland) and were sterile-filtered with 0.22 µm pore size sterile disposable syringe filters. 

#### 3.7.2. Cell Culture

HaCaT cell line (the normal immortalised human keratinocytes) used in the study derived from the 35th passage (the original material created by Prof. Dr. Petra Boukamp and Dr. Norbert Fusenig [68]) was purchased from Cell Line Service GmbH (Eppelheim, Germany). The cells were cultured and propagated as described previously [69,70,71].

#### 3.7.3. MTT (3-(4,5-Dimethylthiazol-2-yl)-2,5-Diphenyltetrazolium Bromide) Assay

The cytotoxicity was assessed with MTT assay according to methodology applied previously [72,73].

The following final concentrations (in four replicates per experiment) of the test compounds were tested (µg/mL): 3.9; 7.8; 15.6; 31.1; 62.2; 124.5; 248.9; 497.8, and 995.6. The final concentration of DMSO in each tested sample did not exceed 0.5% (it was not cytotoxic for cells). Three independent experiments were conducted. The vehicle was cells in the culture medium. The assay was conducted for 24 h and 48 h.

### 3.8. Antibacterial Activity

The antimicrobial activity of the HESP, ligands (HHSB, HIN, and HTSC), and complexes with HP-β-CD were tested against Gram-positive bacteria (*Listeria monocytogenes* ATCC 19111, *Listeria monocytogenes* ATCC 19112, *Listeria monocytogenes* ATCC 19115, *Staphylococcus aureus* ATCC 29737, *Staphylococcus aureus* ATCC 25923, *Staphylococcus aureus* ATCC 27734) and Gram-negative bacteria (*Escherichia coli* ATCC 11303, *Escherichia coli* ATCC 35218, *Salmonella* Typhimurium, ATCC 14028, *Salmonella* Enteritidis ATCC 13076, *Salmonella* Choleraesuis ATCC 7001). The strains were activated from cryobanks by passaging them twice onto a Nutrient Broth (Merck, Darmstadt, Germany). All bacteria were grown for 24 h at 37 °C. Antagonistic activity of the following chemical compounds: HESP, HTSC, HHSB, HIN, HTSC/HP-β-CD, HP-β-CD, HTSC/HP-β-CD (mech), HHSB/HP-β-CD, HHSB/HP-β-CD (mech), H/HP-β-CD, H/HP-β-CD (mech), HIN/HP-β-CD, and HIN/HP-β-CD (mech) were determined by the diffusion-well method. The test strains were plated on Mueller–Hinton Agar (Merck, Darmstadt, Germany) in the form of a surface layer of bacteria according to the procedures recommended by the European Committee on Antimicrobial Susceptibility Testing EUCAST [74].

The detailed methodology was described in the paper by Sykuła et al. [75]. Bacterial inoculum was prepared from an overnight solid culture. The inoculum density was 1–2 × 108 CFU/mL, which corresponds to a McFarland standard of 0.5.

The samples of the test compounds were dissolved in 5% (*w*/*w*) DMSO to obtain a concentration of 500 μM, and were sterilized using filtration (filter pore width 0.2 μm; (Sartorius AG, Gottingen, Germany).

The bacteria inoculum test culture (0.1 mL) smear conducted was on sterile Mueller–Hinton Agar (Merck, Darmstadt, Germany) plates. Wells with a diameter of 8 mm were cut in the medium. A total of 50 µL of the prepared solutions of the tested compounds at a concentration of 500 µM were added to the wells. The plates prepared in this way were incubated at 37 °C for 18 h. After this time, zones of growth inhibition were read and the result was given in mm. The experiment was carried out in two independent repetitions.

The results were expressed in average values and standard deviation. The results were analyzed using a one-way ANOVA and post-hoc Tukey’s test.

#### Minimal Inhibition Concentration (MIC)

The MIC was performed for compounds and strains sensitive to their action in the antagonistic test. Minimum inhibitory concentration was determined by combining the dilution method with the diffusion-well method. Test compounds at a concentration of 500 μM were prepared in 5% (*w*/*w*) DMSO. Surface layer of microorganisms, against which antagonistic activity was noted, were prepared in the same way as for the antagonistic test. Then, two-fold sequential dilutions of the test compounds in the range of 500 µM to 62.5 µM were prepared. Wells with a diameter of 8 mm cut in the Mueller–Hinton Agar (Merck, Darmstadt, Germany) with the test strain were added with 50 µL of the prepared dilutions, which corresponded to the following concentrations of the tested compounds: 2.5 µM, 1.25 mM, 0.625 mM, and 0.3125 µM. Petri dishes prepared in this way were incubated at 37 °C for 18 h. After a given time, the MIC value was read. The MIC concentration expressed in µM was the first concentration of the tested compounds at which growth inhibition of the test strain was observed.

## 4. Conclusions

Compounds extracted from plants exhibit phenomenal biological activities, for example, carotenoids, fat soluble vitamins, phytosterols, polyunsaturated lipids, curcuminoids, and flavonoid compounds. However, putting them into products such as food, cosmetics, or drugs poses some solubility problems. The solubility of active compounds is a crucial parameter in their future application. Due to their poor solubility in water, the activity is rather limited.

Current research work describes the effect of HP-β-CD cyclodextrin for encapsulating hesperetin Schiff bases. All synthesized systems were characterized by various spectro-analytical techniques, including Fourier transform infrared spectroscopy (FT-IR), scanning electron microscopy (SEM), differential scanning calorimetry (DSC) for solid mixtures/systems studies, and ultraviolet–visible spectroscopy (UV-Vis) and nuclear magnetic resonance spectroscopy (NMR) studies of mixtures/systems in aqueous solution. Biological studies, such as cytotoxic and antibacterial activities and antioxidant activity using DPPH^•^ and ABTS^•+^ scavenging ability and Fe^2+-^chelating ability, were also used in the present work. The inclusion systems were prepared using mechanical (mech) and co-evaporation (CV) methods using a molar ratio compound: HP-β-CD equal 1:1. The identification of solid systems of hesperetin and its derivatives with HP-β-CD confirmed the formation of two inclusion complexes of hesperetin (CV) and HHSB (mech). The identification of systems of hesperetin and its derivatives with HP-β-CD in solutions at pHs 3.6, 6.5, and 8.5 and at various temperatures (25, 37, and 60 °C) confirmed the effect of cyclodextrin on their solubility and related implications. In DPPH^•^ and ABTS^•+^ assay, pure compounds were characterized by higher antioxidant activity than the complexes. In the FRAP study, all hesperetin and HHSB complexes and HTSC/HP-β-CD (mech) were characterized by higher values of antioxidant activity than pure compounds. The results obtained from cytotoxic activity tests show that for most of the systems tested, cytotoxicity increased with the concentration of the chemical, with the exception of HP-β-CD. All systems inhibited *Escherichia coli* ATCC 11303 and *Staphylococcus aureus* ATCC 25923.

Our study should be considered significant; however, further research is needed to obtain inclusion complexes for other hesperetin derivatives.

## Figures and Tables

**Table 1:** The chemical shifts of HP-β-CD and hesperetin, HHSB, HIN, and HTSC/ HP-β-CD systems.

	δ [ppm]						
	H1	H2	H3	H4	H5	H6	H-Me
HP-β-CD	5.0582	3.6362	3.9353	3.5774	3.8558	3.8558	1.1316
H/HP-β-CD-(mech)	5.0617	3.6367	3.9341	3.5803	3.8187	3.8535	1.1367
H/HP-β-CD	5.0603	3.6366	3.9304	3.5757	3.8230	3.8505	1.1362
HHSB/HP-β-CD-(mech)	5.0550	3.6363	3.9276	3.5748	3.8483	3.8483	1.1282
HHSB/HP-β-CD	5.0555	3.6374	3.9294	3.5494	3.8489	3.8489	1.1297
HTSC/HP-β-CD-(mech)	5.0545	3.6327	3.9316	3.5732	3.8521	3.8521	1.1271
HTSC/HP-β-CD	5.0684	3.6418	3.9463	3.5629	3.8661	3.8661	1.1419
HIN/HP-β-CD-(mech)	5.0517	3.6301	3.9293	3.5720	3.8490	3.8490	1.1243
HIN/HP-β-CD	5.0685	3.6468	3.9432	3.5858	3.8637	3.8637	1.1418

**Table 2:** Aqueous solubility (S_0_), complexation constant (K_c_), complexation efficiency (CE), and molar ratio of hesperetin and its Schiff bases (HTSC, HHSB, and HIN) at different pH values with HP-β-CD. In brackets, the solubility values of the compounds without the presence of cyclodextrin HP-β-CD are included.

		pH 3.6				pH 6.5				pH 8.5			
Temp.	Compound/HP-β-CD	S_0_ [mM]	K_c_ [M^−1^]	CE [%]	Molar Ratio	S_0_ [mM]	K_c_ [M^−1^]	CE [%]	Molar Ratio	S_0_ [mM]	K_c_ [M^−1^]	CE [%]	Molar Ratio
25 °C	Hesp	0.108 ± 0.004 (0.05 ± 0.002)	3221 ± 358	34.7	1:30	0.208 ± 0.11 (0.049 ± 0.002)	11933 ± 1949	248	1:1	0.32 ± 0.032 (0.28 ± 0.002)	2513 ± 143	80.4	1:3
	HTSC	0.054 ± 0.03 (0.017 ± 0.006)	13 ± 2.9	0.07	1:1430	0.061 ± 0.020 (0.011 ± 0.004)	11 ± 1.6	0.07	1:1431	0.07 ± 0.03 (0.02 ± 0.001)	12.5 ± 0.3	0.1	1:1001
	HHSB	0.041 ± 0.021 (0.018 ± 0.008)	341 ± 79	1.4	1:72	0.045 ± 0.005 (0.025 ± 0.004)	150 ± 21	0.7	1:144	0.045 ± 0.005 (0.025 ± 0.004)	150 ± 19	0.7	1:144
	HIN	0.068 ± 0.04 (0.029 ± 0.009)	102 ± 43	0.7	1:144	0.052 ± 0.024 (0.017 ± 0.003)	39 ± 3.6	0.2	1:501	0.09 ± 0.05 (0.02 ± 0.007)	143 ± 1.6	1.3	1:78
37 °C	Hesp	0.14 ± 0.04 (0.08 ± 0.008)	3171 ± 887	44.4	1:3	0.110 ± 0.03 (0.085 ± 0.001)	2280 ± 152	25	1:5	0.11 ± 0.01 (0.098 ± 0.005)	1703 ± 163	19	1:6
	HTSC	0.076 ± 0.035 (0.028 ± 0.02)	16 ± 1.7	0.1	1:1001	0.068 ± 0.033 (0.027 ± 0.025)	16 ± 1.7	0.1	1:1001	0.16 ± 0.054 (0.22 ± 0.003)	18 ± 4.2	0.3	1:334
	HHSB	0.05 ± 0.01 (0.02 ± 0.01)	226 ± 92	1.1	1:88	0.041 ± 0.023 (0.005 ± 0.004)	618 ± 36	2.5	1:40	0.015 ± 0.0061 (0.005 ± 0.005)	100 ± 34	0.15	1:668
	HIN	0.09 ± 0.05 (0.02 ± 0.006)	145 ± 13	1.3	1:78	0.015 ± 0.009 (0.001 ± 0.0025)	25 ± 3.9	0.04	1:2501	0.014 ± 0.01(0.001 ± 0.003)	28 ± 3.9	0.04	1:2501
60 °C	Hesp	0.13 ± 0.02 (0.095 ± 0.002)	2566 ± 144	34	1:4	0.096 ± 0.009 (0.082 ± 0.001)	1179 ± 888	11	1:1	0.15 ± 0.027 (0.12 ± 0.002)	2365 ± 221	37	1:4
	HTSC	0.17 ± 0.065 (0.037 ± 0.002)	28 ± 0.3	0.5	1:201	0.18 ± 0.06 (0.16 ± 0.05)	2.2 ± 0.6	0.04	1:251	0.27 ± 0.089 (0.25 ± 0.006)	3.2 ± 0.6	0.1	1:1001
	HHSB	0.036 ± 0.014 (0.012 ± 0.002)	250 ± 20	0.9	1:112	0.115 ± 0.053 (0.027 ± 0.002)	155 ± 4	1.8	1:57	0.096 ± 0.042 (0.018 ± 0.0009)	728 ± 82	7	1:15
	HIN	0.041 ± 0.02 (0.008 ± 0.002)	62 ± 1.7	0.3	1:334	0.041 ± 0.02 (0.008 ± 0.002)	61 ± 2.6	0.3	1:334	0.283 ± 0.027 (0.143 ± 0.1)	132 ± 23	3.7	1:28

**Table 3:** IC_50_ values (µg/mL) of tested chemical compounds after 24 and 48 h exposure of human keratinocyte HaCaT.

Chemical Compound	IC_50_ [µg/mL]	
	After 24 h	After 48 h
Hesperetin	220.9	150.0
HTSC	Nd ^a^	11.9
HHSB	417.5	163.5 ^b^
HIN	350.4	59.7
HP-β-CD	nd	nd
H/HP-β-CD	nd	842.9
H/HP-β-CDmech	nd	nd
HTSC/HP-β-CD	710.8	160.4
HTSC/HP-β-CDmech	351.7	182.7
HHSB/HP-β-CD	166.7	12.3
HHSB/HP-β-CDmech	347.3	9.0
HIN/HP-β-CD	862.9	530.3
HIN/HP-β-CDmech	364.8	239.6

^a^ nd—not detected; ^b^ IC_70._

**Table 4:** Antagonistic activity of selected compounds at a concentration of 2.5 µM expressed as the zone of growth inhibition of the test microorganism in [mm].

	Compounds	Zone of Inhibition [mm]												
Strains		Hesperetin	HTSC	HHSB	HIN	HP-β-CD	H/HP-β-CD	H/HP-β-CD-m	HTSC/HP-β-CD	HTSC/HP-β-CD-m	HHSB/HP-β-CD	HHSB/HP-β-CD-m	HIN/HP-β-CD	HIN/HP-β-CD-m
*Escherichia coli* ATCC 11303		9.5 ^ac^ ± 0.71	0.0 ^b^ ± 0.00	0.0 ^b^ ± 0.00	9.0 ^a^ ± 0.00	10.0 ^ac^ ± 0.00	10.0 ^ac^ ± 0.00	10.0 ^ac^ ± 0.00	10.0 ^ac^ ± 1.41	10.0 ^ac^ ± 0.00	9.0 ^ac^ ± 0.00	11.0 ^c^ ± 0.00	9.0 ^a^ ± 0.00	9.0 ^a^ ± 0.00
*Escherichia coli* ATCC 35218		0.0 ^b^ ± 0.00	0.0 ^b^ ± 0.00	9.0 ^a^ ± 0.00	0.0 ^b^ ± 0.00	0.0 ^b^ ± 0.00	0.0 ^b^ ± 0.00	0.0 ^b^ ± 0.00	0.0 ^b^ ± 0.00	9.0 ^a^ ± 0.00	0.0 ^b^ ± 0.00	0.0 ^b^ ± 0.00	0.0 ^b^ ± 0.00	0.0 ^b^ ± 0.00
*Listeria monocytogenes* ATCC 19111		0.0 ± 0.00	0.0 ± 0.00	0.0 ± 0.00	0.0 ± 0.00	0.0 ± 0.00	0.0 ± 0.00	0.0 ± 0.00	0.0 ± 0.00	0.0 ± 0.00	0.0 ± 0.00	0.0 ± 0.00	0.0 ± 0.00	0.0 ± 0.00
*Listeria monocytogenes* ATCC 19112		0.0 ± 0.00	0.0 ± 0.00	0.0 ± 0.00	0.0 ± 0.00	0.0 ± 0.00	0.0 ± 0.00	0.0 ± 0.00	0.0 ± 0.00	0.0 ± 0.00	0.0 ± 0.00	0.0 ± 0.00	0.0 ± 0.00	0.0 ± 0.00
*Listeria monocytogenes* ATCC 19115		0.0 ± 0.00	0.0 ± 0.00	0.0 ± 0.00	0.0 ± 0.00	0.0 ± 0.00	0.0 ± 0.00	0.0 ± 0.00	0.0 ± 0.00	0.0 ± 0.00	0.0 ± 0.00	0.0 ± 0.00	0.0 ± 0.00	0.0 ± 0.00
*Staphylococcus aureus* ATCC 29737		0.0 ^c^ ± 0.00	10.0 ^ab^ ± 0.00	0.0 ^c^ ± 0.00	0.0^c^ ± 0.00	12.0 ^a^ ± 0.00	9.0 ^ab^ ± 0.00	0.0 ^c^ ± 0.00	10.0 ^ab^ ± 1.41	9.5 ^b^ ± 0.71	10.0 ^ab^ ± 0.00	0.0 ^c^ ± 0.00	0.0 ^c^ ± 0.00	12.0 ^a^ ± 0.00
*Staphylococcus aureus* ATCC 25923		0.0 ^a^ ± 0.00	9.0 ^bd^ ± 0.00	12.0 ^c^ ± 0.00	11.0 ^bcd^ ± 0.00	8.5 ^d^ ± 0.71	9.0 ^bd^ ± 0.00	9.0 ^bd^ ± 0.00	8.5 ^d^ ± 0.71	9.5 ^bd^ ± 0.71	10.0 ^bcd^ ± 1.41	9.0 ^bd^ ± 0.00	9.5 ^bd^ ± 0.71	9.0 ^bd^ ± 0.00
*Staphylococcus aureus* ATCC 27734		0.0 ± 0.00	0.0 ± 0.00	0.0 ± 0.00	0.0 ± 0.00	0.0 ± 0.00	0.0 ± 0.00	0.0 ± 0.00	0.0 ± 0.00	0.0 ± 0.00	0.0 ± 0.00	0.0 ± 0.00	0.0 ± 0.00	0.0 ± 0.00
*Salmonella* Typhimurium ATCC 14028		0.0 ± 0.00	0.0 ± 0.00	0.0 ± 0.00	0.0 ± 0.00	0.0 ± 0.00	0.0 ± 0.00	0.0 ± 0.00	0.0 ± 0.00	0.0 ± 0.00	0.0 ± 0.00	0.0 ± 0.00	0.0 ± 0.00	0.0 ± 0.00
*Salmonella* Enteritidis ATCC 13076		0.0 ± 0.00	0.0 ± 0.00	0.0 ± 0.00	0.0 ± 0.00	0.0 ± 0.00	0.0 ± 0.00	0.0 ± 0.00	0.0 ± 0.00	0.0 ± 0.00	0.0 ± 0.00	0.0 ± 0.00	0.0 ± 0.00	0.0 ± 0.00
*Salmonella* Choleraesuis ATCC 7001		0.0 ± 0.00	0.0 ± 0.00	0.0 ± 0.00	0.0 ± 0.00	0.0 ± 0.00	0.0 ± 0.00	0.0 ± 0.00	0.0 ± 0.00	0.0 ± 0.00	0.0 ± 0.00	0.0 ± 0.00	0.0 ± 0.00	0.0 ± 0.00

Statistical analysis: one-way ANOVA, post-hoc Tukey’s test within strain (horizontal), *p* ≤ 0.005. ^a,b,c,d^ statistical differences within one microorganism.

**Table 5:** Minimum concentration of the studied compounds (in µM) that inhibited the growth of test microorganisms.

	Compounds	MIC [µM]												
Strains		H	HTSC	HHSB	HIN	HP-β-CD	H/HP-β-CD	H/HP-β-CD mech	HTSC/HP-β-CD	HTSC/HP-β-CD-mech	HHSB/HP-β-CD	HHSB/HP-β-CD mech	HIN/HP-β-CD	HIN/HP-β-CD-mech
*Escherichia coli* ATCC 11303		2.5	nd	nd	2.5	2.5	2.5	2.5	1.25	1.25	2.5	2.5	2.5	2.5
*Escherichia coli* ATCC 35218		nd	nd	2.5	nd	nd	nd	nd	nd	2.5	nd	nd	nd	nd
*Staphylococcus aureus* ATCC 29737		nd	2.5	nd	nd	2.5	2.5	nd	2.5	2.5	2.5	nd	nd	2.5
*Staphylococcus aureus* ATCC 25923		nd	1.25	2.5	2.5	2.5	2.5	2.5	2.5	1.25	2.5	2.5	2.5	2.5

nd—not determined.

## Data Availability

Not applicable.

## Supplementary Materials

The following supporting information can be downloaded at: https://www.mdpi.com/article/10.3390/molecules28196893/s1, Table S1: Selected experimental peaks (in cm^−1^) and band assignment of hesperetin, HP-β-CD, physical mixture and mixture; Table S2: Selected experimental peaks (in cm^−1^) and band assignment of HHSB, HP-β-CD, physical mixture and mixture; Table S3: Selected experimental peaks (in cm^−1^) and band assignment of HIN, HP-β-CD, physical mixture and mixture; Table S4: Selected experimental peaks (in cm^−1^) and band assignment of HTSC, HP-β-CD, physical mixture and mixture. Figure S1. Cytotoxicity of tested chemicals after (A) 24 and (B) 48 h of exposure of HaCaT cells (human keratinocyte) in MTT (3-(4,5-Dimethylthiazol-2-yl)-2,5-Diphenyltetrazolium Bromide) assay. Each point represents the mean absorbance values of the four replicates from three independent experiments (±standard deviation of the mean—S.E.M.) [35,36,37,38,39,40,41,45].

## Sample Availability

Available.
