# Supplementary material for: Encapsulation and Biological Activity of Hesperetin Derivatives with HP-β-CD
Source: Molecules. 2023 Sep 30;28(19):6893. doi: 10.3390/molecules28196893 (PMC10574185; doi:10.3390/molecules28196893)
Supplement: Supplementary file 1 [file molecules-28-06893-s001.zip › molecules-2611219-supplementary.pdf]

Supplementary materials

Encapsulation and Biological Activity of Hesperetin Derivatives with HP-β-CD

Anna Sykuła [1], Agnieszka Bodzioch [2], Adriana Nowak [3], Waldemar Maniukiewicz [4], Sylwia Ścieszka [5],
Lidia Piekarska-Radzik [5], Elżbieta Klewicka [5], Damian Batory [6] and Elżbieta Łodyga-Chruścińska [1,*]

[1] Faculty of Biotechnology and Food Sciences, Institute of Natural Products and Cosmetics, Lodz University of Technology, Stefanowskiego 2/22, 90-537 Lodz, Poland; anna.sykula@p.lodz.pl
[2] Centre of Molecular and Macromolecular Studies Polish Academy of Sciences, Sienkiewicza 112,
90-363 Lodz, Poland; agnieszka.bodzioch@cbmm.lodz.pl
[3] Department of Environmental Biotechnology, Faculty of Biotechnology and Food Sciences, Lodz University of Technology, Wólczańska 171/173, 90-530 Lodz, Poland; adriana.nowak@p.lodz.pl
[4] Faculty of Chemistry, Institute of General and Ecological Chemistry, Lodz University of Technology, Żeromskiego 116, 90-924 Lodz, Poland; waldemar.maniukiewicz@p.lodz.pl
[5] Institute of Fermentation Technology and Microbiology, Faculty of Biotechnology and Food Sciences, Lodz University of Technology, 90-530 Lodz, Poland; sylwia.scieszka@p.lodz.pl (S.Ś.);
lidia.piekarska-radzik@p.lodz.pl (L.P.-R.); elzbieta.klewicka@p.lodz.pl (E.K.)
[6] Department of Vehicles and Fundamentals of Machine Design, Lodz University of Technology, 90-924 Lodz, Poland; damian.batory@p.lodz.pl
* Correspondence: elzbieta.lodyga-chruscinska@p.lodz.pl

Table S1. Selected experimental peaks (in cm^{-1}) and band assignment of hesperetin and HP-β-CD.

Hesperetin (H)	HP-β-CD	H/HP-β-CD CV	H/HP-β-CD mech	Band assignment
3496			3493	O-H s at A and B ring [1]
	3362	3330	3335	O-H s [2]
	2971	2971	2960	CH_3 and CH vibrations [3,4]
2956				C-H s at B ring
2937				C-H s at B ring
	2922	2926	2926	C–H bonds in the CH and CH_2 groups [2,4,5]
2910				C-H s in methoxy group at B ring [6]
	2884	2885	2878	Vibration of the -CH and -CH_2-groups
2876				C-H symmetric s in methylene group at C ring
2838				C-H symmetric s in methoxy group at B ring
1634		1641	1637	C=O s at C ring + C-C-C asymmetric s in A ring [2]
1610			1610	
1578		1588	1581	C-C-C sc in A ring + C-O-H sc at A ring
1503		1509	1501	C-C-C s in B ring + C-O-H sc, C-H w in methyl group and C-H r at B ring
1475		1494	1462	C-O-H sc + C-H r at A ring + C-C s in A ring + C-H r at C ring
	1458	1462		asymmetric C–H deformation vibrations in the plane
1442			1440	C-C-C s in Bi ring + C-H r + C-O-H sc + C-H w in methyl group and C-H r at B ring
1401			1398	Breathing B ring + C-C-C asymmetric s in A ring + C-H w at C ring + C-H w in methylene group at C ring
	1365	1364		C–H vibration [4,5]
1360			1360	C–H r + C–H w in methylene group at C ring + C–C–C s in B ring + –O–H sc at B ring
1338			1338	breathing B ring + C–C–C s in A ring + C–H r in all molecule
1305			1306	C–H w + C–H w in methylene group at C ring + –O–H sc at B ring
1283				C–H w at C ring + C–H r + –O–H sc + –C–O s at B ring
1260			1262	C–H w at C ring + breathing A ring + C–H r and –O–H sc at A ring
1241		1243	1241	C–H w at ring C + C–H r and –O–H r at A and B ring
1201			1204	–O–H r at A and B ring + C–H r at B ring + C–H w at C ring

1188		1186		
1169				breathing B ring + C–H w and C–H t in methylene group at C ring + –O–H r at A ring
	1150	1158	1151	coupled vibration of C–O, C–O–C, C–C–O and C–C–C asymmetric valence vibrations [4]
1125		1123	1127	C–C–C sc in B ring + C–H sc and O–H r at B ring
1093				C–O–C assymetric s in C ring + C–C–C r in A ring + –O–H r at A ring
	1082	1079	1081	coupled vibration of C–O, C–O–C, C–C–O and C–C–C asymmetric valence vibrations [4]
1066				C–C s in C ring
1027		1025	1024	def. B ring + –O–C– s in methoxy group at B ring + C–C–C s in A ring
	1017			coupled vibration of C–O, C–O–C, C–C–O and C–C–C asymmetric valence vibrations [4]
955				breathing A ring + def. C ring + C–C–C s in B ring + C–H r at B ring
	947	948	947	the presence of glucopyranose units of 2-hydroxypropyl-β-cyclodextrin in C1 chair conformation [2]
877		874		C–H w at B ring
	856	853		α-type glycosidic bond [4]
813		820	812	C–H w at A and B ring
	756	755	758	
736		710	712	C–H w at A ring
650		657	650	def. all molecule

Abbreviations: def. -deformation, r-rocking, s-stretching, sc-scissoring, t-twisting, w-wagging

Table S2. Selected experimental peaks (in cm^{-1}) and band assignment of HHSB and HP-β-CD.

HHSB	HP-β-CD	HHSB/HP-β-CD CV	HHSB/HP-β-CD mech	Band assignment
3614				N-H s
3497				O-H s at A and B ring [1]
	3362	3342	3316	O-H s [4]
3314				N-H s [6]
3181				O-H s
3054				O-H s
3007				C-H s in methoxy group at B ring
	2971	2970	2973	CH_3 and CH vibrations [3]
	2922	2930	2929	C–H bonds in the CH and CH_2 groups [5]
	2884			Vibration of the -CH and -CH_2-groups
1691			1689	
1640		1640	1640	C=O s
1614		1604	1614	
1540		1527	1542	C-C-C sc in A ring + C-O-H sc at A ring
1511		1509	1514	C-C-C s in B ring + C-O-H sc, C-H w in methyl group and C-H r at B ring
1486			1487	C-O-H sc + C-H r at A ring + C-C s in A ring + C-H r at C ring
	1458	1458	1460	asymmetric C–H deformation vibrations in the plane
1455				C-C-C s in B ring + C-H r + C-O-H sc + C-H w in methyl group and C-H r at B ring
		1407		

1397				Breathing B ring + C-C-C asymmetric s in A ring + C-H w at C ring + C-H w in methylene group at C ring
	1365			C–H vibration [5]
1355			1354	C–H r + C–H w in methylene group at C ring + C–C–C s in B ring + –O–H sc at B ring
1292			1299	C–H w + C–H w in methylene group at C ring + –O–H sc at B ring
		1278		C–H w at C ring + C–H r + –O–H sc + –C–O s at B ring
1264				C–H w at C ring + breathing A ring + C–H r and –O–H sc at A ring
1222				C–H w at ring C + C–H r and –O–H r at A and B ring
1155	1150	1150	1153	coupled vibration of C–O, C–O–C, C–C–O and C–C–C asymmetric valence vibrations
1123			1123	C–C–C sc in B ring + C–H sc and O–H r at B ring
	1082	1075	1072	coupled vibration of C–O, C–O–C, C–C–O and C–C–C asymmetric valence vibrations
1067				C–C s in C ring
		1021	1020	
1014				def. B ring + –O–C– s in methoxy group at B ring + C–C–C s in A ring
	1017			coupled vibration of C–O, C–O–C, C–C–O and C–C–C asymmetric valence vibrations
956				breathing A ring + def. C ring + C–C–C s in B ring + C–H r at B ring
	947	946	949	the presence of glucopyranose units of 2-hydroxypropyl-β-cyclodextrin in C1 chair conformation
871				C–H w at B ring
	856	855	837	hydrogen bond formation beetwen primary and secondary OH group [1] and the presence of glucopyranose units of HP-β-CD in C1 chair conformation
810		810	810	C–H w at A and B ring
760	756	760	759	C-H b
707		706	712	C–H w at A ring
650		650	652	def. all molecule

Abbreviations: b – bending, def. -deformation, r-rocking, s-stretching, sc-scissoring, t-twisting, w-wagging,

Table S3. Selected experimental peaks (in cm^{-1}) and band assignment of HIN and HP-β-CD.

HIN	HP-β-CD	HIN/HP-β-CD CV	HIN/HP-β-CD mech	Band assignment
3479		3474		O-H s at A and B ring [1]
	3362		3338	O-H s [4]
3314				N-H s [7]
3206		3204		O-H s
3091				C=O [
3007				C-H s in methoxy group at B ring
	2971		2968	CH_3 and CH vibrations [3]
	2922		2929	C–H bonds in the CH and CH_2 groups [5]
	2884			Vibration of the -CH and -CH_2-groups

1698			1699	
1662		1660	1664	C=O
1634		1637	1636	C=N
1599		1598	1596	pyridine
1546				C-C-C sc in A ring + C-O-H sc at A ring
1516		1517	1518	C-C-C s in B ring + C-O-H sc, C-H w in methyl group and C-H r at B ring
	1458	1455	1457	asymmetric C–H deformation vibrations in the plane
1454		1413	1411	Pyridine [8]
1363	1365	1365	1366	C–H vibration [5]
1313		1310		C–H r + C–H w in methylene group at C ring + C–C–C s in B ring + –O–H sc at B ring
1276		1277	1280	C–H w at C ring + C–H r + –O–H sc + –C–O s at B ring
1236		1235	1237	C–H w at ring C + C–H r and –O–H r at A and B ring
1186	1150	1186	1187	coupled vibration of C–O, C–O–C, C–C–O and C–C–C asymmetric valence vibrations
1160		1160	1159	C–C–C sc in B ring + C–H sc and O–H r at B ring
1092	1082	1092	1079	coupled vibration of C–O, C–O–C, C–C–O and C–C–C asymmetric valence vibrations
1069		1070		C–C s in C ring
1019		1021	1022	def. B ring + –O–C– s in methoxy group at B ring + C–C–C s in A ring
	1017			coupled vibration of C–O, C–O–C, C–C–O and C–C–C asymmetric valence vibrations
972		976		breathing A ring + def. C ring + C–C–C s in B ring + C–H r at B ring
	947	946	947	the presence of glucopyranose units of 2-hydroxypropyl-β-cyclodextrin in C1 chair conformation
876		875		C–H w at B ring
	856	846	847	hydrogen bond formation between primary and secondary OH group and the presence of glucopyranose units of HP-β-CD in C1 chair conformation
789		791	790	C–H w at A and B ring
752	756	755	757	C-H b
736		732	735	C–H w at A ring
662		645	665	def. all molecule

Abbreviations: b – bending, def. -deformation, r-rocking, s-stretching, sc-scissoring, t-twisting, w-wagging,

Table S4. Selected experimental peaks (in cm^{-1}) and band assignment of HTSC and HP-β-CD.

HTSC	HP-β-CD	HTSC/HP-β-CD CV	HTSC/HP-β-CD mech	Band assignment
3440		3441	3441	O-H s at A and B ring [1]

	3362			O-H s [4]
3329		3329	3328	N-H s [7]
			3211	O-H s
3065				C=O [
3016				C-H s in methoxy group at B ring
2933	2971	2974		CH_3 and CH vibrations [3]
	2922		2930	C–H bonds in the CH and CH_2 groups [5]
1641		1645	1644	C=N
1598		1602	1601	
1534		1537	1536	C-C-C sc in A ring + C-O-H sc at A ring
1503		1516	1513	C-C-C s in B ring + C-O-H sc, C-H w in methyl group and C-H r at B ring
	1458		1481	asymmetric C–H deformation vibrations in the plane
1474				
1460		1459	1462	
1442			1444	
		1409		
		1379		
1359	1365	1361	1360	C–H vibration [5] [J Incl Phenom Macrocycl Chem (2016) 86:7–1]
		1335		
1272		1277	1276	C=S
1212		1208		C–H w at ring C + C–H r and –O–H r at A and B ring
	1150			coupled vibration of C–O, C–O–C, C–C–O and C–C–C asymmetric valence vibrations
1163				C–C–C sc in B ring + C–H sc and O–H r at B ring
1128			1126	
	1082	1080		coupled vibration of C–O, C–O–C, C–C–O and C–C–C asymmetric valence vibrations
1067			1068	C–C s in C ring
1024		1020	1022	def. B ring + –O–C– s in methoxy group at B ring + C–C–C s in A ring
	1017			coupled vibration of C–O, C–O–C, C–C–O and C–C–C asymmetric valence vibrations
953				breathing A ring + def. C ring + C–C–C s in B ring + C–H r at B ring

	947	946		the presence of glucopyranose units of 2-hydroxypropyl-β-cyclodextrin in C1 chair conformation
924				
898			898	C–H w at B ring
870		870	869	
860				
	856	857	855	hydrogen bond formation between primary and secondary OH group [1] and the presence of glucopyranose units of HP-β-CD in C1 chair conformation
840		839	839	
809		813	811	C–H w at A and B ring
765		765	761	C-H b
	756	757		
679				C–H w at A ring
664		655	664	def. all molecule

Abbreviations: b – bending, def. -deformation, r-rocking, s-stretching, sc-scissoring, t-twisting, w-wagging.

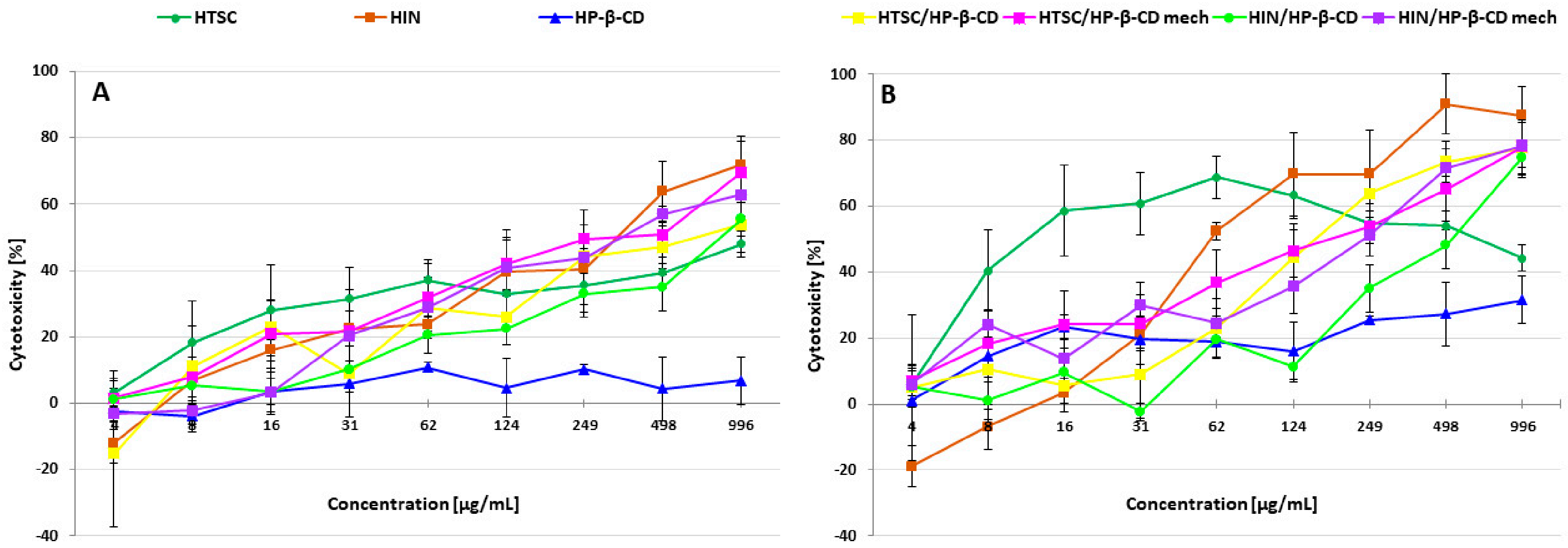

Figure S1. Cytotoxicity of tested chemicals after (**A**) 24 and (**B**) 48 h exposition of HaCaT cells (human keratinocyte) in MTT (3-(4,5-Dimethylthiazol-2-yl)-2,5-Diphenyltetrazolium Bromide) assay. Each point represents the mean absorbance values of the four replicates from three independent experiments (± standard deviation of the mean – S.E.M.).

1. Wdowiak, K.; Rosiak, N.; Tykarska, E.; Żarowski, M.; Płazińska, A.; Płaziński, W.; Cielecka-Piontek, J. Amorphous Inclusion Complexes: Molecular Interactions of Hesperidin and Hesperetin with HP-B-CD and Their Biological Effects. *Int J Mol Sci* **2022**, *23*, doi:10.3390/ijms23074000.

2. Zoghbi, A.; Geng, T.; Wang, B. Dual Activity of Hydroxypropyl-β-Cyclodextrin and Water-Soluble Carriers on the Solubility of Carvedilol. *AAPS PharmSciTech* **2017**, *18*, 2927–2935, doi:10.1208/s12249-017-0769-2.

3. Kim, J.-S. Study of Flavonoid/Hydroxypropyl-β-Cyclodextrin Inclusion Complexes by UV-Vis, FT-IR, DSC, and X-Ray Diffraction Analysis. *Prev Nutr Food Sci* **2020**, *25*, 449–456, doi:10.3746/pnf.2020.25.4.449.

4. Yuan, C.; Liu, B.; Liu, H. Characterization of Hydroxypropyl-β-Cyclodextrins with Different Substitution Patterns via FTIR, GC–MS, and TG–DTA. *Carbohydr Polym* **2015**, *118*, 36–40, doi:10.1016/j.carbpol.2014.10.070.

5. Savic-Gajic, I.; Savic, I.M.; Nikolic, V.D.; Nikolic, L.B.; Popsavin, M.M.; Kapor, A.J. Study of the Solubility, Photostability and Structure of Inclusion Complexes of Carvedilol with β-Cyclodextrin and (2-Hydroxypropyl)-β-Cyclodextrin. *J Incl Phenom Macrocycl Chem* **2016**, *86*, 7–17, doi:10.1007/s10847-016-0635-y.

6. Colthup B.Norman; Daly H. Lawrence; Wiberley E. Stephen *Introduction to Infrared and Raman Spectroscopy (Third Edition)*; Academic Press, 1990;

7. Shi, Y.; Zhang, T.; Ren, H.; Kruse, A.; Cui, R. Polyethylene Imine Modified Hydrochar Adsorption for Chromium (VI) and Nickel (II) Removal from Aqueous Solution. *Bioresour Technol* **2018**, *247*, 370–379, doi:10.1016/j.biortech.2017.09.107.

8 Barzetti, T.; Selli, E.; Moscotti, D.; Forni, L. Pyridine and Ammonia as Probes for FTIR Analysis of Solid Acid Catalysts. *Journal of the Chemical Society, Faraday Transactions* **1996**, *92*, 1401, doi:10.1039/ft9969201401.
